# Associations of genome-wide structural variations with phenotypic differences in cross-bred Eurasian pigs

**DOI:** 10.1186/s40104-023-00929-x

**Published:** 2023-10-07

**Authors:** Wencheng Zong, Jinbu Wang, Runze Zhao, Naiqi Niu, Yanfang Su, Ziping Hu, Xin Liu, Xinhua Hou, Ligang Wang, Lixian Wang, Longchao Zhang

**Affiliations:** 1 State Key Laboratory of Animal Biotech Breeding, Institute of Animal Sciences, Chinese Academy of Agricultural Sciences, Beijing, 100193 China; 2 College of Animal Science, Shanxi Agricultural University, Jinzhong, 030801 China; 3 College of Animal Science and Technology, Qingdao Agricultural University, Qingdao, 266109 China

**Keywords:** Body size, GWAS, Pig, Skeleton, Structural variations

## Abstract

**Background:**

During approximately 10,000 years of domestication and selection, a large number of structural variations (SVs) have emerged in the genome of pig breeds, profoundly influencing their phenotypes and the ability to adapt to the local environment. SVs (≥ 50 bp) are widely distributed in the genome, mainly in the form of insertion (INS), mobile element insertion (MEI), deletion (DEL), duplication (DUP), inversion (INV), and translocation (TRA). While studies have investigated the SVs in pig genomes, genome-wide association studies (GWAS)-based on SVs have been rarely conducted.

**Results:**

Here, we obtained a high-quality SV map containing 123,151 SVs from 15 Large White and 15 Min pigs through integrating the power of several SV tools, with 53.95% of the SVs being reported for the first time. These high-quality SVs were used to recover the population genetic structure, confirming the accuracy of genotyping. Potential functional SV loci were then identified based on positional effects and breed stratification. Finally, GWAS were performed for 36 traits by genotyping the screened potential causal loci in the F2 population according to their corresponding genomic positions. We identified a large number of loci involved in 8 carcass traits and 6 skeletal traits on chromosome 7, with *FKBP5* containing the most significant SV locus for almost all traits. In addition, we found several significant loci in intramuscular fat, abdominal circumference, heart weight, and liver weight, etc.

**Conclusions:**

We constructed a high-quality SV map using high-coverage sequencing data and then analyzed them by performing GWAS for 25 carcass traits, 7 skeletal traits, and 4 meat quality traits to determine that SVs may affect body size between European and Chinese pig breeds.

**Supplementary Information:**

The online version contains supplementary material available at 10.1186/s40104-023-00929-x.

## Background

Genome rearrangements generate an abundance of structural variations (SVs) that, despite occurring mainly in non-coding regions, can determine the binding of transcriptional regulatory elements, mRNA splicing and processing, genome folding and higher order structures, and translational alterations due to their size and location [1, 2]. In general, SVs can be divided into two types based on changes in the DNA content of the genome: 1) unbalanced copy number variants (CNVs), including deletions (DELs), duplications (DUPs), insertions (INSs), and mobile element insertions (MEIs); and 2) balanced rearrangements, including inversions (INVs) and translocations (TRAs) [3]. In livestock research, SVs have been shown to be associated with adaptability and production traits [4, 5]. Compared to SNPs, SVs contribute to a higher proportion of complex phenotypes [6]. As a consensus, SVs are defined as a significant mutational force shaping genome evolution and function [7].

During the long process of domestication and selection, which began about 10,000 years ago in Europe (Near East) and Asia (China), pig breeds with independent biological traits and breed-specific genomic variants have emerged [8]. The Large White pig is a common Western commercial breed with a long carcass, fast growth rate, high lean meat ratio, and high feed utilization efficiency [9]. In contrast, Min pigs distributed in Northeastern China perform relatively poorly for these traits, but show better tolerance to harsh conditions, roughage, and have high intramuscular fat [10]. The construction of reference populations has become an important method for determining the associations between genomic variants and phenotypic differences in agricultural research, and genome-wide association studies (GWAS) have provided a wealth of new information in the last decade [11, 12]. Here, an F2 population was constructed based on a cross between 4 Large White and 15 Min pigs, which were selected for traits according to Mendel's law of free association and provided an opportunity to subsequently study phenotypic differences.

In recent years, several SV studies on pig genome have been reported [13–16]. Nevertheless, GWAS based on SVs are rarely reported, which limits our understanding of the potential function and exploitation of SVs as genetic markers. Previous SV studies were performed at a low sequencing depth, which may reduce the sensitivity and accuracy of SV identifications. As a consensus, most SV studies rely on multiple software to increase the number and accuracy of SV identifications, but this approach is usually computationally resource-intensive and time-consuming in population-scale studies. Moreover, most SV-calling software does not recognize insertion variants or subsequently does not genotype them accurately, which has led most SV studies to ignore the contribution of insertion variants to animal phenotypes.

In this context, a high-quality SV map containing 4 SV types was constructed using resequencing data with an average depth of more than 35×. Subsequently, potential causal loci that were screened between breeds were rapidly genotyped in a large population of offspring according to our own designed strategy. Finally, we performed GWAS for 36 traits to determine the effect of SVs on phenotype. To our knowledge, this may be the most comprehensive trait association study of the pig genome using SV markers to date. In conclusion, our study provides a new strategy for SV research at the population scale that has been demonstrated to be reliable and efficient. Meanwhile, this study will provide a new theoretical basis for using SVs as molecular markers or developing marker-assisted selection and deepen the understanding of the potential function of SVs in the pig genome.

## Methods

### Animal collection

The pigs used in the experiment were all from the Large White × Min pig resource population, raised at the Changping pig farm of the Institute of Animal Sciences, Chinese Academy of Agricultural Sciences. The Large White × Min pig resource population included 19 F0 individuals and 513 F2 individuals, among which the F0 individuals included 4 Large White and 15 Min pigs. The 15 Min pigs were collected from the Jilin Academy of Agricultural Sciences (JAAS) and the rural areas of Northeastern China, and the 4 Large White pigs were from the United Kingdom. All F2 individuals were raised to market age (240 ± 7 d) and slaughtered for commercial purposes. Paired-end sequencing was performed using Illumina Hi-seq 2500, with a sequencing depth of > 30× for F0 individuals and 5–7× for F2 individuals. The sequencing data of the F0 and F2 individuals used in this study have been submitted to the Genome Sequence Archive (GSA) with the accession number CRA002451. We downloaded data for additional 11 Large White pigs from the NCBI Sequence Read Archive (SRA, https://www.ncbi.nlm.nih.gov/bioproject/?term=PRJEB39374), and information for all individuals is shown in Table S1, Additional file 1.

### Phenotype determination

The F2 population constructed with Large White and Min pigs collected abundant quantitative traits. For the convenience of this study, we divided all the traits into 25 carcass traits, 7 skeletal traits, and 4 meat quality traits. Among them, carcass traits include carcass length, body length, body height, cannon circumference, scapular width, chest width, chest depth, abdominal circumference, waist width, hip width, hip length, hip circumference, bone rate, total weight of front bone, total weight of middle bone, total weight of hind bone, total weight of front lean meat, total weight of middle lean meat, total weight of hind lean meat, total weight of front fat, total weight of middle fat, total weight of hind fat, heart weight, liver weight, and lung weight, skeletal traits include scapula length, humerus length, forearm bone length, hip bone length, femur length, calf bone length, and vertebral number, and meat quality traits include marbling, intramuscular fat, tenderness, and moisture percentage. All phenotypic characteristics were defined according to the Animal Genetic Resources in China (pig) [10], Wikipedia (https://en.wikipedia.org), published breed genetic resource studies, and the official websites of pig breeds.

### Data processing

The raw reads were trimmed with Trimmomatic [17], and high-quality trimmed reads were aligned against the pig reference genome (Sscrofa11.1) with Bwa [18]. Samtools [19] was employed to convert sam files to bam format and subsequently sort and index the bam files. The PCR duplicates were marked with Picard [20]. Samples that had more than one associated bam file were merged with samtools. The sequencing depth statistics of all samples were calculated using mosdepth [21].

### SV calling, filtering, and validation

The primary research approach was to employ several software to detect SVs, essential to maximize the obtained SV loci. Thus, five SV software were selected for SV discovery: Delly v0.9.1, Smoove v0.2.8, Manta v1.6.0, Breakdancer v1.4.5, and MELT v2.2.2. Among them, Delly, Smoove, Manta, and Breakdancer were used to identify DELs, DUPs, and INVs, whereas MELT was used to identify MEIs. Previous studies have relied on the overlap of results from several different SV software, although this strategy does not reliably improve detection and may even aggravate false discoveries [22]. In this study, we merged the results of several software analyses instead of including only overlaps, to maximize the number of SV loci obtained. Among these software, Smoove, Delly, Breakdancer, and Manta called SVs according to default parameters. For MELT, Repeatmasker [23] was employed to annotate the pig reference genome for MEI using the RepeatMasker libraries (2018-10-26) of Repbase database (https://www.girinst.org/), and the three most widely distributed types of ERV, LINE, and SINE were selected for MEI calls, and the reference sequences are displayed in Table S2. Survivor [24] software was used to merge the SV datasets of each software, which were defined and merged according to a distance of 1,000 bp between breakpoints, taking into account the strands and types of SVs. Subsequently, all individuals were merged to generate the SV map. Genotyping of DELs, DUPs, and INVs was performed using SVtyper [25], whereas that of MEIs was performed using MELT.

To control the quality of all SV loci, we also set a strict filter for each SV locus, keeping only loci with QUAL > 200 for DELs, DUPs, and INVs and only loci with "PASS" for MEIs, to ensure the quality of each SV locus. For INVs, variants within 1,000 bp could not be merged because of the reverse position of breakpoints output by different software. We merged the loci with more than 75% overlap between each breakpoint using our own written script. Meanwhile, these loci were divided into two groups, < 100 kb and > 100 kb, and merged separately in order to avoid large variants covering small variants. For SVs of 1–10 Mb, we used Samplot [26] for visualization and randomly selected three loci of each SV type for PCR validation to assess accuracy. The primer design schematics and primer sequence information for all SV types are shown in Fig. S1 (Additional file 2) and Table S3 (Additional file 1) respectively. To test the accuracy of SV genotyping, we randomly selected one SV locus per chromosome and verified them by PCR. For DEL, DUP, and MEI, we determined the primers based on around 500 bp of the breakpoints on both sides of the SV locus, whereas for INV, one primer is set about 500 bp upstream or downstream of the SV breakpoint and the other primer is set inside the INV. PCR was then performed using DNA from 19 F0 individuals, and the primer sequences are listed in Table S4, Additional file 1.

### Population genetics, SV functional, and *F*_ST_ analysis

The sample geographic distribution map was produced using the ggplot2 [27] and ggspatial packages in R. Principal component analysis (PCA) was performed using the GCTA [28] software. Population structure was evaluated using Admixture [29] and three possible populations (K = 2–4) were calculated. Next, the ggplot2 package was employed to plot the PCA and population structure results. The neighbor-joining trees were constructed using Phylip [30] and visualized by MEGA11 [31]. SV distribution locations were determined based on gene location annotations from the Ensemble database, and SV effects were estimated by Snpeff [32] based on the locations of SV breakpoints. In order to identify the breed-stratified SVs, the VCFtools [33] was used to calculate the fixation index (*F*_ST_) values of all SVs with the Weir and Cockerham method comparing 15 Min vs. 15 Large White pigs. All SVs within the differentiated region with *F*_ST_ values in the top 5% were selected, and the corresponding genes overlapping with SVs were considered as candidates for breed stratification based on the gene information annotated by Ensemble. The candidate genes were annotated using Gene Ontology (GO) and Kyoto Encyclopedia of Genes and Genomes (KEGG) functional enrichment analysis by DAVID online webloci (https://david.ncifcrf.gov/). The wordcloud webloci (https://www.jasondavies.com/wordcloud/) was used to create word clouds for the top 10 significantly enriched terms (*P* < 0.05) to reveal underlying molecular mechanisms.

### Population-specific SV screening, genotyping, and GWAS

In order to obtain potential causal loci affecting specific traits, a screening strategy for differentially genotyped loci was used. Six relatively purebred Min pigs (M1, M3–M7) and 15 Large White pigs were selected for this screening strategy. Two conditions were set: first, one genotype was selected for one breed and the remaining two genotypes were set for the other breed; second, the frequency of genotypes was more than 80% for Min pigs (≥ 5 individuals) and more than 50% for Large White pigs (≥ 8 individuals) (Fig. S2, Additonal file 2). An SV locus was considered a candidate locus when it matched the above two conditions. The screened SV loci were genotyped using the bam file of the F2 individuals, among which, DELs, DUPs, and INVs were genotyped using SVtyper, whereas MEIs were genotyped using paragraph [34]. Plink [35] was performed to filter all genotyped SV loci with the following specific parameters: a sample call rate of > 90%, an SV call rate of > 90%, and a minor allele frequency of > 5%. EMMAX [36] was employed to perform GWAS using a mixed linear model for all filtered SV loci. The sex and slaughter batch were used as fixed effects, and the PCA was used as a covariate. The significance cutoff was defined as the Bonferroni test threshold, which was set as 0.05/(total number of SVs). All GWAS results were visualized using the rMVP [37] package.

## Result

### The landscape-wide SV discovery in the genomes of the Large White and Min pig

We developed an SV study for the Large White × Min pig resource population, which included 19 F0 individuals and 513 F2 individuals. Among them, the F0 individuals involved 4 Large White and 15 Min pigs. In order to obtain a more comprehensive SV landscape, we collected additional 11 Large White pigs (PRJEB39374). Then, whole-genome resequencing data were obtained for the 15 Large White and 15 Min pigs, with a total of approximately 1,396 G data obtained after quality control and an average sequencing depth of more than 35×. The sequencing data were aligned to the Sscrofa11.1 using BWA-mem, and SVs were called by Delly [38], Manta [39], Smoove (https://github.com/brentp/smoove), Breakdancer [40], and MELT [41]. All variants from the results of each SV tool were merged individually and then further merged at the population level based on SV breakpoint locations, types, and orientations, thus generating an initial SV dataset. After quality control, manual merging, and genotyping to produce the final SV landscape, a detailed SV analysis pipeline was created, which is presented in Fig. S3, Additional file 2. To exclude false positives generated from NGS data due to length limitations, most previous studies limited the SV length to 50 bp–10 Mb [13, 14, 42], but recent study used a range of 50 bp–1 Mb [43]. In this study, we visualized all loci from 1–10 Mb using Samplot and randomly selected three loci from DELs, DUPs, and INVs for PCR validation. We found that all SVs in the range of 1–10 Mb did not show the expected bands, and the genomic coverage map could not determine whether variants were present (Fig. S4–S6, Additional file 2). This result showed that SVs in the range of 1–10 Mb were not reliable, so loci > 1 Mb were excluded from this analysis. In addition, to assess the quality of the SVs identified, one locus per chromosome was selected randomly for PCR analysis using all F0 individuals. The results showed that almost all loci showed bands of the expected size with an accuracy of 94.46% (Fig. S7, Additional file 2).

**Fig. 1:**
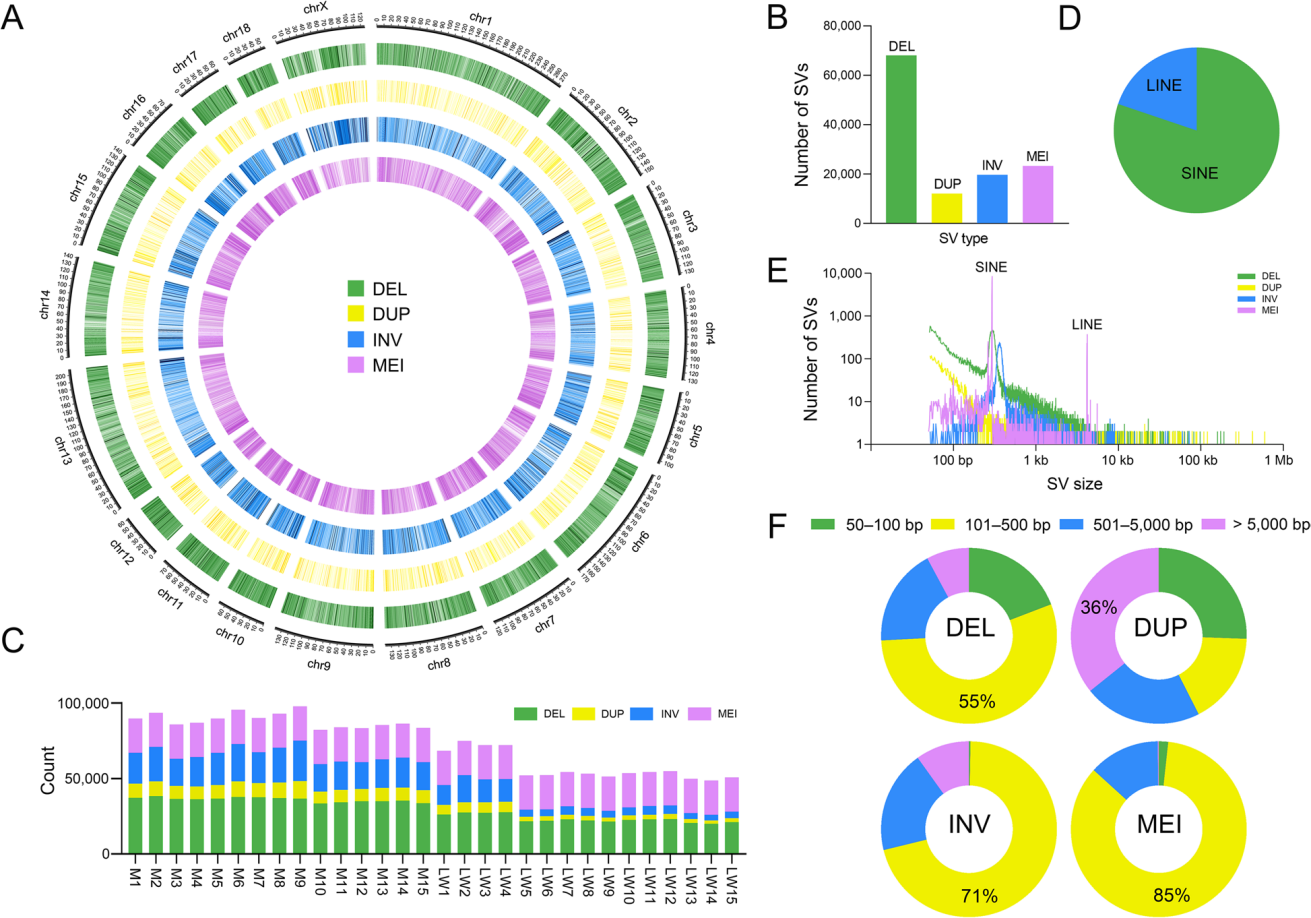
The SV landscape of Large White and Min pigs. **A** Distribution of the discovered SVs in the pig genome. The circle diagram shows the distribution of SVs in the chromosomes, where concentric circles show the following from outside to inside: DELs, DUPs, INVs, and MEIs. **B** Total number of SVs identified per type. Statistics on the number of loci per SV type in the final generated SV dataset. **C** Number of each SV type for 15 Large White and 15 Min pigs. Stacked bar graph showing the number of SVs initially called for each sample, containing 18 autosomes and X chromosome. **D** Percentage of transposon types in MEIs. The pie chart shows the percentage statistics of ERV, LINE, and SINE transposons. **E** SV size distribution per SV type with *x*-axis and *y*-axis shown in log10 scale. **F** Distribution of length range per SV type. Four length ranges are labeled above the doughnut chart

A high-quality landscape with 123,151 SV loci, including 68,121 DELs, 12,045 DUPs, 19,727 INVs, and 23,258 MEIs (Fig. 1A and B) was obtained from the analysis. We compared the identified SV loci with the Ensemble public SV database (version 22-08-26) and filtered them with a 75% overlap rate, and a total of 66,435 new SV loci were found, which greatly enriched the public SV database (Fig. S8, Additional file 2). This result suggests that the sensitivity of each software differs for different genomic regions and that combining the results of multiple software for identifying SVs and using high-coverage sequencing data would significantly increase the number of new SV loci discovered. Among the 19 F0 individuals, more SVs were identified in Min pigs than in Large White pigs (Fig. 1C and Table S5, Additional file 1). Compared to European pigs, the genomes of Chinese local pig breeds confers higher genetic diversity [44]. In addition, fewer SVs were identified in the remaining 11 Large White pigs (LW5–LW15) than in the 19 F0 individuals, which may be related to the sequencing depth (Fig. 1C). SINEs and LINEs contributed to all MEI types (Fig. 1D), with SINEs accounting for more than 80% of the major insertion types. Notably, SINEs have previously been reported to contribute a large number of polymorphisms in the pig genome [45]. We further investigated the size distribution of the identified SVs with length between 50 bp and 1 Mb. Most SVs were small (< 500 bp), with a large number of SINEs and LINEs identified as the variant size increased (Fig. 1E and F). The DELs, INVs, and MEIs were mainly within the range of 100–500 bp, while the DUPs were mainly large SVs of > 5,000 bp (Fig. 1F and Table S6, Additional file 1).

### Population structure inference

**Fig. 2:**
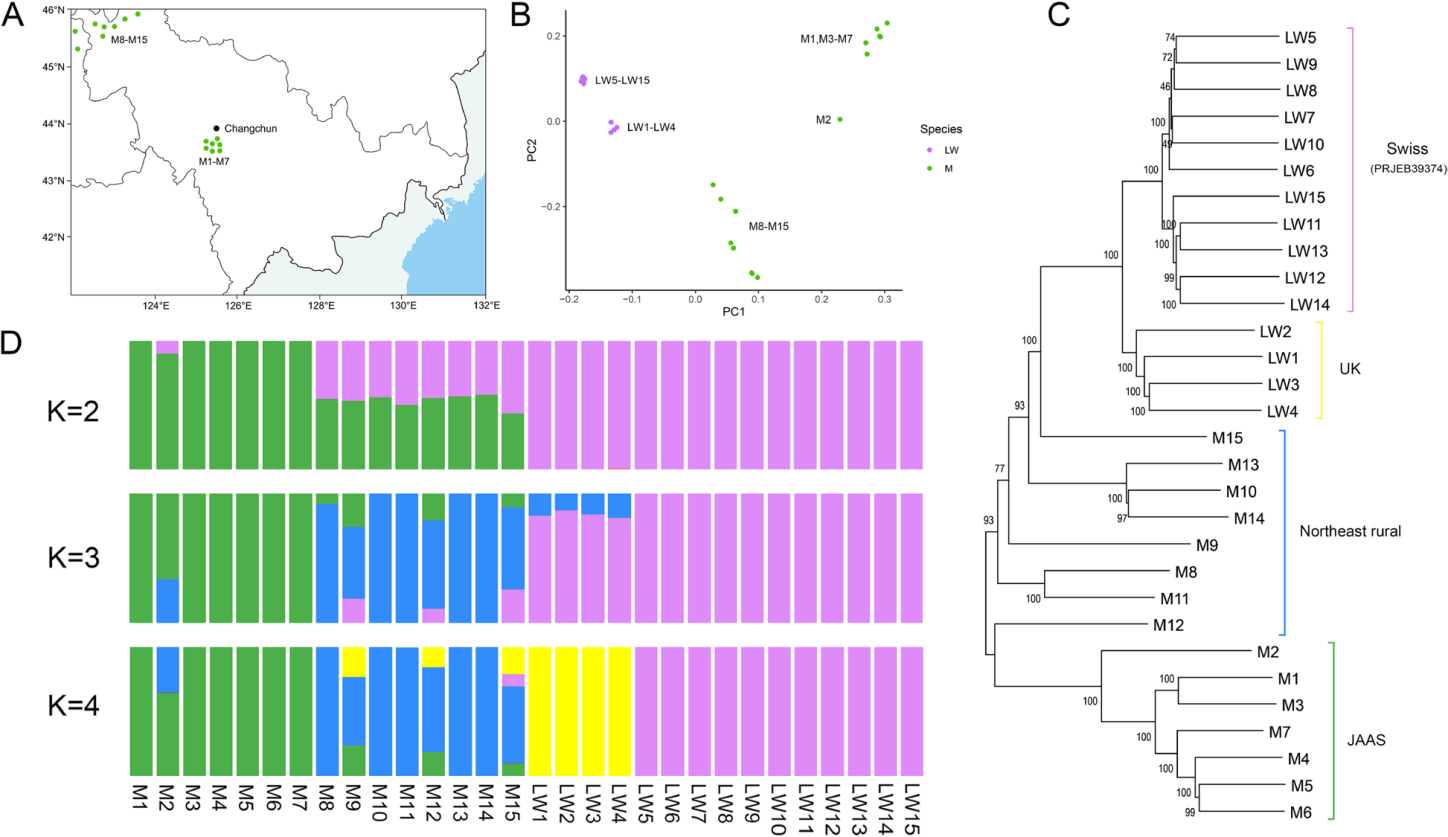
Population genetic analysis using SV markers. **A** The geographical distribution of Min pigs used in this study. **B** PCA derived from SVs. Purple represents Large White pigs and green represents Min pigs. **C** Phylogenetic tree constructed for Large White and Min pigs based on whole-genome SV data. Green represents Min pigs from JAAS, blue represents Min pigs from rural areas of Northeast China, yellow represents Large White pigs from the UK, and purple represents Large White pigs from Swiss. **D** Genome-wide admixture analyses inferred from SVs (K = 2, 3, and 4). Each individual is a vertical rectangle with different colors implying different genetic populations

To further confirm data quality, we used the discovered SVs to infer the population genetic structure of 15 Large White and 15 Min pigs (Fig. 2A–D). PCA was performed uniformly for all SV genotypes. The results confirmed the separation of the Min pigs into distinct groups from the Large White pigs (Fig. 2B). Depending on the sampling location (Fig. 2A), the Min pigs from the JAAS and those from other areas in Northeast China also imply different pedigrees. In addition, 4 Large White pigs of F0 generation (UK) and 11 Large White pigs from Swiss (PRJEB39374) are also clearly separated. The above results, which are approximately the same as the results of the 50 k chip analysis, reconfirm the accuracy of SV genotyping (Fig. S9, Additional file 2). We also constructed a phylogenetic tree, which can be divided into four clusters (Fig. 2C): the first and second clusters are Swiss and UK Large White pigs, respectively, and the fourth cluster is JAAS Min pigs. The third cluster does not form a single cluster, implying that these individuals have a complex pedigree. Therefore, we further executed a population structure analysis (Fig. 2D) and found that the Min pigs (M8–M15) sampled in rural areas of Northeastern China may have undergone crossbreeding, showing a clear exotic bloodline infiltration. This may be due to the introduction of commercial pig breed pedigrees by local people to improve economic efficiency.

### Functional relevance of SVs

**Fig. 3:**
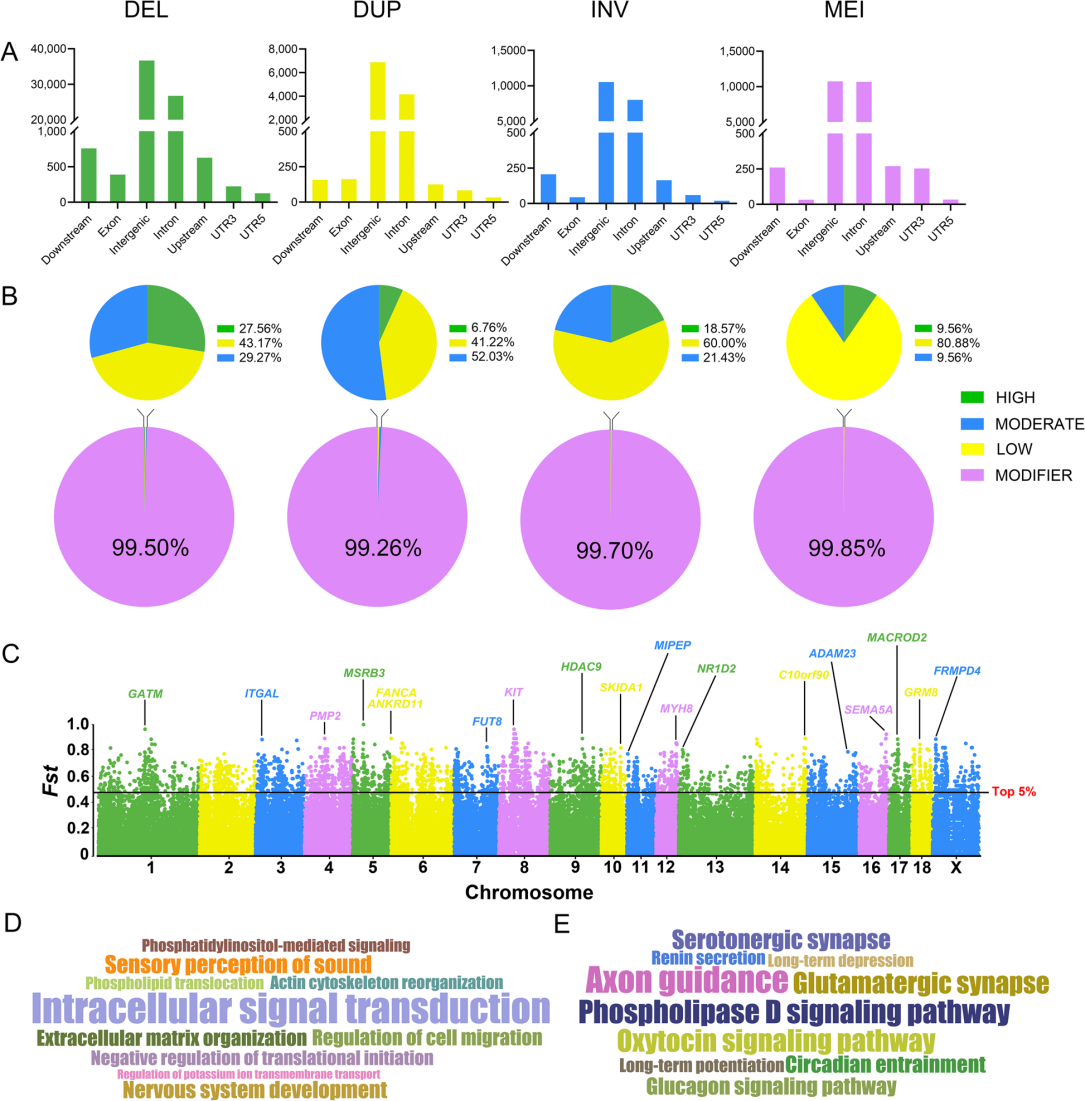
Position effect estimation and *F*_ST_ screening. **A** Distribution of the location per SV type in the genome. The *x*-axis is the genomic positions and the *y*-axis is the number of SVs. **B** Proportion of effects predicted for each SV type. Predicted effects based on SV distribution locations in the pig genome were, in order, "MODIFIER", "LOW", "MODERATE", and "HIGH". **C** Manhattan plot based on Weir and Cockerham's fixed index (*F*_ST_) statistics. The nearest gene of the SV locus with the highest *F*_ST_ value was marked on each chromosome. **D** GO and **E** KEGG enrichment analysis based on the top 5% of *F*_ST_ loci overlapping genes. The font size of GO terms and KEGG pathways correlates with the number of enriched genes

To explore the potential functions of SVs, we investigated their locations in the genome, including gene downstream, exon, intergenic, intron, gene upstream, and untranslated regions (UTR3 and UTR5). All four SV types are located mainly at intergenic and intron positions, with DELs, DUPs, INVs, and MEIs accounting for 96.75%, 95.15%, 97.40%, and 96.18%, respectively (Fig. 3A). The remaining SVs were located in the coding region, the untranslated region, and within 1 kb upstream and downstream of the gene. Approximately 42.40% of SVs overlapped, with one or more Ensemble genes. The different types of SVs did not show a statistically specific preference for positional distribution, implying that the distribution of SVs was independent of the SV type.

We further predicted the effects for four SV types according to their distribution in the genome. Most of the SV effects were defined as "MODIFIER", implying they generally had no effect on genes (Fig. 3B). The remaining SV effects were defined as "HIGH", "MODERATE", and "LOW". Among them, the proportion of DELs, DUPs, INVs, and MEIs with "HIGH" effects was 27.56%, 6.76%, 18.57%, and 9.56%, respectively (Fig. 3B and Table S7, Additional file 1). The SVs with "HIGH" effects were annotated, which revealed that they involve several disease-related pathways, including Coronavirus disease-COVID-19 (ssc05171), Parkinson's disease (ssc05012), Non-alcoholic fatty liver disease (ssc04932), and Alzheimer disease (ssc05010) (Fig. S10, Additional file 2).

### Breed-stratified SVs

In order to discover candidate adaptive SVs, we calculated the *F*_ST_ between 15 Large White and 15 Min pigs. The top 5% were identified as potential breed-stratified SV loci with a total of 3,797 DELs, 271 DUPs, 231 INVs, and 525 MEIs (Fig. 3C and Table S8, Additional file 1). We annotated the SV loci with the highest *F*_ST_ values on each chromosome to identify potentially functional genes that may be affected. Among them, the SV locus with the highest *F*_ST_ was *MSRB3* on chromosome 5, which was previously reported to have a key role in pig ear size [46]. *MYH8* has been reported to be associated with muscle development and meat quality traits [47], and *NR1D2* is responsible for adipogenesis and lipid accumulation in the myocardium [48, 49]. The *KIT* locus is a key gene in determining coat color in different pig breeds [50]. *GATM* and *SEMA5A* are involved in placental development and embryonic development, respectively [51, 52]. *HDAC9* and *GRM8* are associated with eye muscle area and the relative area of type I fibers, respectively [53, 54]. *ITGAL* is immune-related and involved in leukocyte recruitment processes [55]. *FANCA* is associated with cell meiosis and germ cell development, and its mutation leads to reduced fertility and follicular reduction [56]. *ADAM23*, *ANKRD11*, and *MACROD2* function in the nervous system and are associated with several neurological disorders [57–61]. *FUT8* disruption leads to growth retardation, early postnatal developmental death, and emphysema-like changes in the lungs [62]. *MIPEP* expression is up-regulated in response to heat stress [63], and *FRMPD4* is highly expressed in pig breeds with high teat numbers [64]. In addition, *SKIDA1* is related to the survival of human embryonic stem cells [65]. Then, the top 5% of SV loci-associated genes were analyzed using GO and KEGG. A total of 4,824 common SV regions overlapped with 1,440 functional genes, contributing to the enriched terms and pathways. The top 10 significant GO terms and KEGG pathways were enriched for cellular processes and biological regulation, as well as for pathways associated with nervous system function and the endocrine system (Fig. 3D and E). We found that most breed-stratified SVs focused on neurological-related pathways, which may emphasize a special role of these pathways in the domestication and selection of Large White and Min pigs.

### GWAS found that SVs were mainly associated with the body size difference between Large White and Min pigs

**Fig. 4:**
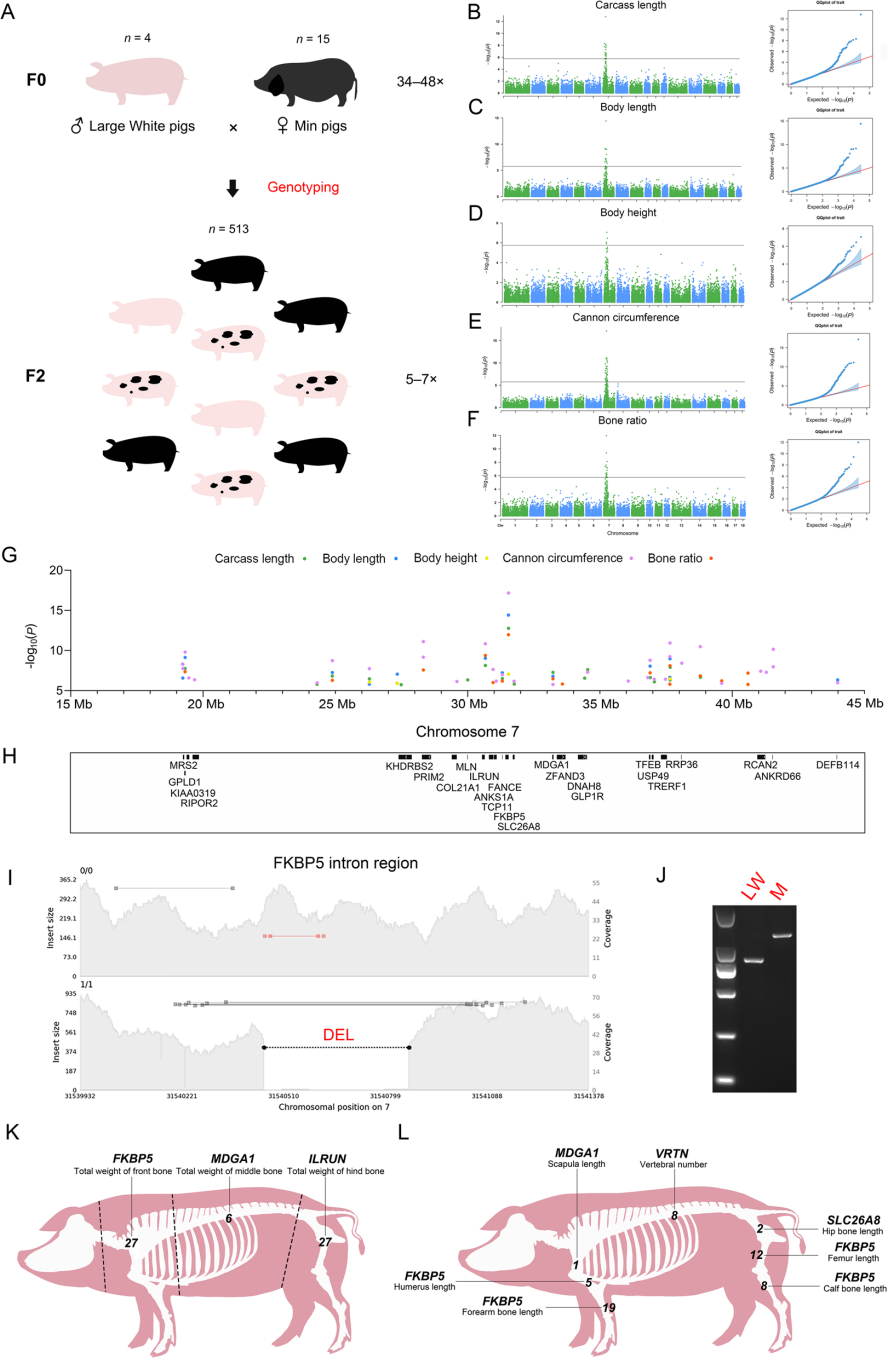
SV-based GWAS. **A** Schematic diagram of Large White × Min pig resource population. The numerals on the top of the pig image represent the number of samples, and the numerals on the right represent the range of sequencing depth. **B–F** Manhattan and quantumquantum (QQ) plots of associated SVs for carcass length (**B**), body length (**C**), body height (**D**), cannon circumference (**E**), and bone rate (**F**). **G** and **H** Manhattan plot of five carcass phenotypes of significant loci at 15–45 Mb on chromosome 7 with corresponding protein-coding genes. **I** The result of genome coverage visualization for *FKBP5* intron region (chr7:31,539,932–31,541,378). The left vertical coordinate shows the insert size of the reads, and the right vertical coordinate shows the genome coverage. The black dotted line marks the location of DEL. **J** Electropherogram of the DEL in the *FKBP5 *intronic region. Electropherogram showing the results of PCR amplification for the Large White and Min pig. The size of electrophoretic bands was indicated with TaKaRa DL2000. **K** GWAS for bone weight in the front, middle, and hind sections of pig carcass. The dotted line represents the segmentation position of the pig carcass. The genes closest to the most significant loci are labeled above each phenotype, with Arabic numerals representing the number of significant SV loci for each phenotype. **L** GWAS for seven pig skeletal phenotypes. The description of this figure is consistent with **K**

The F2 population data processing resulted in a total of approximately 5,110 G of data at a depth of 5–7× . To identify SV loci with phenotypic variation due to differences between breeds, we performed a locus screen for differential genotypes between Large White and Min pigs, and then selected these SV loci for genotyping in the F2 population based on the corresponding genomic positions (Fig. 4A), as described in Methods. Finally, a total of 33,909 loci were screened, of which 97.15% (16,898,906/17,395,317) were successfully genotyped and then GWAS were performed. Bonferroni's multiple testing method was employed for *P*-value correction, which was defined as 0.05/*n*, where *n* represents the number of SVs for each independent GWAS. A total of 36 traits were involved in the GWAS, including 25 carcass traits, 7 skeletal traits, and 4 meat quality traits.

We found that SVs may have an effect on pig body size, and the GWAS identified overlapping strong association peaks for carcass length, body length, body height, cannon circumference, and bone rate involving 87 significant loci (Fig. 4B–F and Table 1) overlapping with 25 protein-coding genes (Fig. 4G and H, and Table 1), including intron variants, as well as upstream and downstream variants of genes. Among them, a DEL of the intron region located in *FKBP5* is the most significant loci for all five traits (Fig. 4I and J, Fig. S11A, Additional file 2 and Table S9, Additional file 1), and this gene has been reported to be involved in osteoclast differentiation [66, 67]. A SINE insertion upstream of *ILRUN* is also one of the most significant loci (Fig. S11B and Table S9), and this gene has been frequently reported to be associated with human height [68–71] as well as carcass length, body length, and cannon circumference in pigs [72, 73]. A DEL was identified in the intron region of *TFEB* (Fig. S11C and Table S9), *RCAN2* (Fig. S11D and Table S9), and *ANKS1A* (Fig. S11E and Table S9), which have been reported to be associated with osteoblast differentiation [74], osteoblast function [75], and bone mineral density [76], respectively. In addition, a DEL was found upstream of *MRS2* (Fig. S11F and Table S9) and downstream of *GLP1R* (Fig. S11G and Table S9), respectively. Among them, *MRS2* is associated with Mg^2+^ expression, and lower Mg^2+^ levels stimulate osteoclast formation [77]. *GLP1R* plays a key role in bone strength and quality [78]. The GWAS results for the above five traits included multiple genes associated with skeleton, suggesting that SVs may affect skeletal size and thus pig body size. We also performed GWAS on scapular width, chest width, chest depth, abdominal circumference, waist width, hip width, hip length, and hip circumference (Fig. S12A–H, Additional file 2). The results showed no significant SV loci for the traits except two significant loci for abdominal circumference (Fig. S12D), which may imply that these carcass traits are poorly correlated with skeleton.

**Table 1:** Summary of significant SV loci in 36 traits

Phenotype	Chr	Position	Gene name	SV location	Type	Length	*P*-value
Abdominal circumference	1	260,511,205	*CDK5RAP2*	Intron	DEL	283	4.24E-07
	1	271,010,710	*LAMC3*	Intron	DEL	284	5.41E-07
Body height	7	31,540,442	*FKBP5*	Intron	DEL	441	8.75128E-08
	7	37,650,773	LncRNA	Intron	DEL	311	3.3894E-07
	7	26,286,305	-	Intergenic	DEL	291	8.85504E-07
	7	27,338,884	-	Intergenic	DEL	2,446	1.23795E-06
Body length	7	31,540,442	*FKBP5*	Intron	DEL	441	3.78792E-15
	7	19,322,838	*GPLD1*	Intron	DEL	101	7.1424E-10
	7	30,669,698	*ILRUN*	Gene upstream	MEI	294	8.99621E-10
	7	37,650,773	LncRNA	Intron	DEL	311	1.05991E-09
	7	36,897,444	*TFEB*	Intron	DEL	136	8.61888E-09
	7	24,883,903	*ENSSSCG00000061569, ENSSSCG00000001455*	Coverage	DUP	19,228	5.58603E-08
	7	31,311,879	*FANCE*	Gene upstream	DEL	288	5.9659E-08
	7	27,338,884	-	Intergenic	DEL	2,446	8.84893E-08
	7	33,224,291	*MDGA1*	Intron	DEL	75	1.61736E-07
	7	37,641,459	LncRNA	Intron	DEL	127	2.19766E-07
	7	19,232,872	*ENSSSCG00000057632*	Gene upstream	MEI	292	2.62207E-07
	7	43,989,593	*DEFB114*	Intron	DEL	1,677	4.49759E-07
	7	26,286,305	-	Intergenic	DEL	291	1.53065E-06
Bone ratio	7	31,540,442	*FKBP5*	Intron	DEL	441	1.04559E-12
	7	30,669,698	*ILRUN*	Gene upstream	MEI	294	4.02735E-10
	7	37,650,773	LncRNA	Intron	DEL	311	7.68871E-09
	7	28,324,725	*PRIM2*	Intron	INV	388	2.68482E-08
	7	19,322,838	*GPLD1*	Intron	DEL	101	4.31208E-08
	7	36,897,444	*TFEB*	Intron	DEL	136	5.94847E-08
	7	40,599,238	-	Intergenic	DUP	309	6.52153E-08
	7	38,794,225	-	Intergenic	DEL	357	1.46567E-07
	7	33,224,291	*MDGA1*	Intron	DEL	75	3.36078E-07
	7	24,883,903	*ENSSSCG00000061569, ENSSSCG00000001455*	Coverage	DUP	19,228	5.02475E-07
	7	39,605,564	-	Intergenic	DEL	694	5.9881E-07
	7	31,311,879	*FANCE*	Gene upstream	DEL	288	6.393E-07
	7	30,962,999	*ANKS1A*	Intron	DEL	92	9.76289E-07
	7	27,336,953	-	Intergenic	DUP	5,095	1.18774E-06
	7	33,582,502	*ZFAND3*	Intron	DEL	65	1.49752E-06
	7	37,641,459	LncRNA	Intron	DEL	127	1.54425E-06
	7	40,599,295	-	Intergenic	DEL	267	1.70419E-06
Calf bone length	7	38,794,225	-	Intergenic	DEL	357	1.54116E-10
	7	31,540,442	*FKBP5*	Intron	DEL	441	2.16357E-08
	7	19,322,838	*GPLD1*	Intron	DEL	101	2.68006E-08
	7	37,641,459	LncRNA	Intron	DEL	127	3.68605E-08
	7	36,897,444	*TFEB*	Intron	DEL	136	7.87112E-08
	7	19,232,872	*ENSSSCG00000057632*	Gene upstream	MEI	292	4.2437E-07
	7	33,093,365	-	Intergenic	DEL	1,547	4.70256E-07
	7	30,669,698	*ILRUN*	Gene upstream	MEI	294	6.1988E-07
Cannon circumference	7	31,540,442	*FKBP5*	Intron	DEL	441	6.71872E-18
	7	28,324,725	*PRIM2*	Intron	INV	388	7.63587E-12
	7	37,650,773	LncRNA	Intron	DEL	311	1.14929E-11
	7	30,669,698	*ILRUN*	Gene upstream	MEI	294	1.41436E-11
	7	38,794,225	-	Intergenic	DEL	357	3.23829E-11
	7	41,553,724	*ANKRD66*	Intron	DEL	673	7.02795E-11
	7	19,322,838	*GPLD1*	Intron	DEL	101	1.56476E-10
	7	37,641,459	LncRNA	Intron	DEL	127	5.70722E-10
	7	28,324,716	*PRIM2*	Intron	DEL	130	6.7774E-10
	7	36,897,444	*TFEB*	Intron	DEL	136	1.62437E-09
	7	24,883,903	*ENSSSCG00000061569, ENSSSCG00000001455*	Coverage	DUP	19,228	1.80288E-09
	7	38,094,955	*RRP36*	Gene downstream	DEL	309	3.80358E-09
	7	19,232,872	*ENSSSCG00000057632*	Gene upstream	MEI	292	5.08074E-09
	7	41,553,727	*ANKRD66*	Intron	INV	721	1.0657E-08
	7	19,239,684	*MRS2*	Gene upstream	DEL	278	1.77718E-08
	7	26,286,305	-	Intergenic	DEL	291	1.80886E-08
	7	30,962,999	*ANKS1A*	Intron	DEL	92	2.25339E-08
	7	41,078,897	*RCAN2*	Intron	DEL	314	3.8015E-08
	7	34,536,073	*GLP1R*	Gene downstream	DEL	221	4.84358E-08
	7	41,288,870	-	Intergenic	DEL	327	5.08201E-08
	7	31,311,879	*FANCE*	Gene upstream	DEL	288	1.01069E-07
	7	36,811,392	-	Intergenic	DEL	53	2.35974E-07
	7	19,463,307	*KIAA0319*	Intron	DEL	309	2.56879E-07
	7	37,476,936	*TRERF1*	Intron	DEL	294	3.72867E-07
	7	37,055,115	*USP49*	Gene upstream	DEL	304	3.76061E-07
	7	19,678,493	*RIPOR2*	Intron	DEL	308	4.41254E-07
	7	36,074,049	-	Intergenic	DEL	268	6.33459E-07
	7	31,084,916	*TCP11*	Intron	DEL	289	6.50236E-07
	7	31,757,248	*SLC26A8*	Intron	DEL	2,450	6.78635E-07
	7	29,593,390	*COL21A1*	Intron	DEL	316	7.1008E-07
	7	43,989,593	*DEFB114*	Intron	DEL	1677	1.00074E-06
	7	24,311,068	*ENSSSCG00000030901*	Coverage	DUP	31,737	1.05293E-06
	7	39,605,564	-	Intergenic	DEL	694	1.22437E-06
	7	33,224,291	*MDGA1*	Intron	DEL	75	1.70098E-06
Carcass length	7	31,540,442	*FKBP5*	Intron	DEL	441	1.65E-13
	7	19,232,872	*ENSSSCG00000057632*	Gene upstream	MEI	292	4.82488E-09
	7	30,669,698	*ILRUN*	Gene upstream	MEI	294	7.31789E-09
	7	37,650,773	LncRNA	Intron	DEL	311	1.22581E-08
	7	19,322,838	*GPLD1*	Intron	DEL	101	1.74936E-08
	7	34,536,073	*GLP1R*	Gene downstream	DEL	221	2.34715E-08
	7	33,224,291	*MDGA1*	Intron	DEL	75	5.21348E-08
	7	24,883,903	*ENSSSCG00000061569, ENSSSCG00000001455*	Coverage	DUP	19,228	1.51579E-07
	7	38,794,225	-	Intergenic	DEL	357	2.21327E-07
	7	36,897,444	*TFEB*	Intron	DEL	136	2.41208E-07
	7	34,419,953	*DNAH8*	Intron	DEL	302	2.72373E-07
	7	31,311,879	*FANCE*	Gene upstream	DEL	288	3.10433E-07
	7	26,286,305	-	Intergenic	DEL	291	3.37836E-07
	7	30,002,117	*MLN*	Gene downstream	DEL	396	4.64132E-07
	7	37,641,459	LncRNA	Intron	DEL	127	4.78724E-07
	7	37,055,115	*USP49*	Gene upstream	DEL	304	8.04377E-07
	7	31,757,248	*SLC26A8*	Intron	DEL	2,450	1.5249E-06
	7	24,311,068	*ENSSSCG00000030901*	Coverage	DUP	31,737	1.6098E-06
	7	27,489,396	*KHDRBS2*	Intron	DEL	879	1.79273E-06
Femur length	7	31,540,442	*FKBP5*	Intron	DEL	441	1.69288E-11
	7	19,322,838	*GPLD1*	Intron	DEL	101	4.4582E-09
	7	37,641,459	LncRNA	Intron	DEL	127	1.41047E-08
	7	30,962,999	*ANKS1A*	Intron	DEL	92	1.74416E-08
	7	38,794,225	-	Intergenic	DEL	357	3.2897E-08
	7	36,897,444	*TFEB*	Intron	DEL	136	7.98636E-08
	3	125,943,216	*NOL10*	Intron^*^	DEL	252	2.17341E-07
	7	38,094,955	*RRP36*	Gene downstream	DEL	309	6.88085E-07
	7	37,055,115	*USP49*	Gene Upstream	DEL	304	7.03532E-07
	7	33,224,291	*MDGA1*	Intron	DEL	75	7.45747E-07
	3	125,953,211	*NOL10*	Intron^*^	DEL	2,681	7.80005E-07
	7	37,650,773	LncRNA	Intron	DEL	311	8.90967E-07
Forearm bone length	7	31,540,442	*FKBP5*	Intron	DEL	441	7.15594E-12
	7	37,641,459	LncRNA	Intron	DEL	127	1.46387E-10
	7	30,962,999	*ANKS1A*	Intron	DEL	92	3.50128E-10
	7	37,650,773	LncRNA	Intron	DEL	311	1.4709E-09
	7	36,897,444	*TFEB*	Intron	DEL	136	2.61096E-09
	7	37,488,354	*TRERF1*	Intron	DEL	300	3.35499E-09
	7	38,794,225	-	Intergenic	DEL	357	3.63713E-09
	7	19,232,872	*ENSSSCG00000057632*	Gene upstream	MEI	292	4.92423E-09
	7	19,322,838	*GPLD1*	Intron	DEL	101	2.78437E-08
	7	33,224,291	*MDGA1*	Intron	DEL	75	1.06476E-07
	7	30,002,117	*MLN*	Gene downstream	DEL	396	2.4688E-07
	7	19,239,684	*MRS2*	Gene upstream	DEL	278	3.66744E-07
	7	37,261,717	*C6orf132*	Gene upstream	DEL	297	4.22894E-07
	7	34,536,073	*GLP1R*	Gene downstream	DEL	221	6.18251E-07
	7	28,324,716	*PRIM2*	Intron	DEL	130	1.04137E-06
	7	30,669,698	*ILRUN*	Gene upstream	MEI	294	1.09207E-06
	7	24,883,903	*ENSSSCG00000061569, ENSSSCG00000001455*	Coverage	DUP	19,228	1.19239E-06
	7	36,074,049	-	Intergenic	DEL	268	1.19749E-06
	7	37,055,115	*USP49*	Gene upstream	DEL	304	1.76888E-06
Heart weight	3	125,953,211	*NOL10*	Intron^*^	DEL	2,681	5.99072E-07
Hip bone length	7	31,757,248	*SLC26A8*	Intron	DEL	2,450	6.10178E-07
	7	24,311,068	*ENSSSCG00000030901*	Coverage	DUP	31,737	7.92587E-07
Humerus length	7	31,540,442	*FKBP5*	Intron	DEL	441	1.35763E-08
	7	34,536,073	*GLP1R*	Gene downstream	DEL	221	4.81907E-08
	7	19,232,872	*ENSSSCG00000057632*	Gene upstream	MEI	292	1.04481E-07
	7	37,641,459	LncRNA	Intron	DEL	127	2.51234E-07
	1	264,535,617	*DENND1A*	Intron	DEL	311	5.70534E-07
Intramuscular fat	12	57,675,134	*HS3ST3A1*	Intron	DEL	122	8.67028E-07
	12	54,564,984	*CFAP52*	Intron	MEI	291	9.75823E-07
	12	54,407,453	*STX8*	Intron	DEL	302	1.19263E-06
Liver weight	7	19,239,684	*MRS2*	Gene upstream	DEL	278	1.50207E-06
Scapula length	7	33,224,291	*MDGA1*	Intron	DEL	75	2.38035E-07
Total weight of front bone	7	31,540,442	*FKBP5*	Intron	DEL	441	2.25255E-12
	7	30,669,698	*ILRUN*	Gene upstream	MEI	294	5.21713E-12
	7	33,224,291	*MDGA1*	Intron	DEL	75	3.8738E-11
	7	37,641,459	LncRNA	Intron	DEL	127	1.47312E-10
	7	36,897,444	*TFEB*	Intron	DEL	136	5.58282E-10
	7	37,650,773	LncRNA	Intron	DEL	311	1.06535E-09
	7	38,794,225	-	Intergenic	DEL	357	2.91302E-09
	7	24,311,068	*ENSSSCG00000030901*	Coverage	DUP	31,737	4.48617E-09
	7	37,055,115	*USP49*	Gene upstream	DEL	304	1.64413E-08
	7	24,883,903	*ENSSSCG00000061569, ENSSSCG00000001455*	Coverage	DUP	19,228	1.70589E-08
	7	28,324,725	*PRIM2*	Intron	INV	388	2.21799E-08
	7	19,232,872	*ENSSSCG00000057632*	Gene upstream	MEI	292	2.60468E-08
	7	34,419,953	*DNAH8*	Intron	DEL	302	6.0505E-08
	7	36,074,049	-	Intergenic	DEL	268	7.33407E-08
	7	30,962,999	*ANKS1A*	Intron	DEL	92	1.05105E-07
	7	38,094,955	*RRP36*	Gene downstream	DEL	309	1.45388E-07
	7	19,322,838	*GPLD1*	Intron	DEL	101	2.07241E-07
	7	37,261,717	*C6orf132*	Gene upstream	DEL	297	2.68119E-07
	7	37,488,354	*TRERF1*	Intron	DEL	300	4.77823E-07
	7	34,536,073	*GLP1R*	Gene downstream	DEL	221	7.19729E-07
	7	28,324,716	*PRIM2*	Intron	DEL	130	7.81429E-07
	7	30,002,117	*MLN*	Gene downstream	DEL	396	7.89703E-07
	7	40,599,295	-	Intergenic	DEL	267	1.01179E-06
	7	43,989,593	*DEFB114*	Intron	DEL	1,677	1.17865E-06
	7	39,605,564	-	Intergenic	DEL	694	1.31736E-06
	7	27,489,396	*KHDRBS2*	Intron	DEL	879	1.49335E-06
	7	35,332,237	LncRNA	Gene upstream	DEL	306	1.59814E-06
Total weight of front lean meat	7	41,494,812	*PLA2G7*	Intron	MEI	294	6.03091E-07
Total weight of hind bone	7	30,669,698	*ILRUN*	Gene upstream	MEI	294	7.95851E-13
	7	38,794,225	-	Intergenic	DEL	357	3.41618E-11
	7	36,897,444	*TFEB*	Intron	DEL	136	3.51948E-11
	7	31,540,442	*FKBP5*	Intron	DEL	441	9.62978E-11
	7	37,650,773	LncRNA	Intron	DEL	311	9.81726E-11
	7	37,641,459	LncRNA	Intron	DEL	127	2.8606E-10
	7	33,224,291	*MDGA1*	Intron	DEL	75	9.30272E-10
	7	19,232,872	*ENSSSCG00000057632*	Gene upstream	MEI	292	3.80882E-09
	7	37,055,115	*USP49*	Gene upstream	DEL	304	4.22536E-09
	7	34,419,953	*DNAH8*	Intron	DEL	302	4.37384E-09
	7	30,962,999	*ANKS1A*	Intron	DEL	92	1.12907E-08
	7	37,488,354	*TRERF1*	Intron	DEL	300	2.10081E-08
	7	19,322,838	*GPLD1*	Intron	DEL	101	2.53408E-08
	7	31,757,248	*SLC26A8*	Intron	DEL	2,450	2.65487E-08
	7	36,074,049	-	Intergenic	DEL	268	6.61502E-08
	7	34,536,073	*GLP1R*	Gene downstream	DEL	221	8.30738E-08
	7	31,311,879	*FANCE*	Gene upstream	DEL	288	9.31817E-08
	7	37,261,717	*C6orf132*	Gene upstream	DEL	297	2.37267E-07
	7	39,605,564	-	Intergenic	DEL	694	2.55411E-07
	7	30,002,117	*MLN*	Gene downstream	DEL	396	4.61386E-07
	7	24,311,068	*ENSSSCG00000030901*	Coverage	DUP	31,737	4.69345E-07
	7	33,093,365	-	Intergenic	DEL	1547	5.24922E-07
	7	19,463,307	*KIAA0319*	Intron	DEL	309	6.42425E-07
	7	38,094,955	*RRP36*	Gene downstream	DEL	309	7.07833E-07
	7	36,811,392	-	Intergenic	DEL	53	1.32686E-06
	7	28,324,725	*PRIM2*	Intron	INV	388	1.32768E-06
	7	27,338,884	-	Intergenic	DEL	2,446	1.46419E-06
Total weight of middle bone	7	33,224,291	*MDGA1*	Intron	DEL	75	6.19796E-09
	7	30,669,698	*ILRUN*	Gene upstream	MEI	294	1.16594E-07
	7	37,650,773	LncRNA	Intron	DEL	311	1.39685E-07
	7	34,419,953	*DNAH8*	Intron	DEL	302	1.59434E-07
	7	31,540,442	*FKBP5*	Intron	DEL	441	1.89109E-07
	6	158,747,897	*GLIS1*	Intron	DEL	85	1.49265E-06
Vertebral number	7	97,615,896	*VRTN*	Intron	MEI	294	5.41E-11
	1	266,822,698	-	Intergenic	MEI	294	2.51235E-09
	1	268,351,956	*AK1*	Gene downstream	DEL	258	2.35774E-07
	1	268,687,871	*DNM1*	Intron	DEL	60	3.94325E-07
	1	267,960,645	*ZNF79*	Exon	MEI	294	7.2393E-07
	7	97,653,457	*SYNDIG1L*	Gene downstream	DEL	765	8.30871E-07
	1	261,580,807	*ENSSSCG00000005518*	Intron	DEL	291	1.05102E-06
	1	262,031,225	*TTLL11*	Intron	DEL	955	1.51808E-06

^*^Indicates that the variation is mostly located in this region

To verify whether there was position specificity in the effect of SVs on skeleton, we divided the pig carcass into three sections, namely front, middle, and hind, according to the position of 4–5 ribs and the lumbosacral joint after removing the head. We then performed GWAS for total weight of front bone, total weight of middle bone, total weight of hind bone, scapula length, humerus length, forearm bone length, hip bone length, femur length, calf bone length, and vertebral number. The results showed that all three sections of bone weight and six bone length traits showed strong association peaks (Fig. S13A–I, Additional file 2 and Table 1), which almost overlapped with previous GWAS results for carcass length, body length, body height, cannon circumference, and bone rate (Fig. S14, Additional file 2). The 7_31540442 (*P* = 2.25255E-12), 7_33224291 (*P* = 6.19796E-09), and 7_30669698 (*P* = 7.95851E-13) were the most significant SV loci in the three sections, corresponding to the three genes: *FKBP5*, *MDGA1*, and *ILRUN* (Fig. 4K). Moreover, *FKBP5* is the most significant gene for humerus, forearm bone, femur, and calf bone length (Fig. 4L). Regarding vertebral number (Fig. S13J), the most significant locus was the 291 bp intron variant (*P* = 5.41E-11) of *VRTN*, which is consistent with a previous study using SNP markers [79]. We also identified a SINE insertion locus (*P* = 7.2393E-07) in the exonic region of the *ZNF79* gene, which was previously associated with bone mineral density [80]. Further analysis, we checked whether SVs affected tissues other than bone by performing GWAS of total lean and fat weight in the front, middle, and hind sections. The results showed only one significant locus for total weight of front lean meat, with no significant association peaks for the remaining traits (Fig. S15A–F, Additional file 2 and Table 1). This result indicates that SVs mainly involve bone tissue and are poorly associated with other tissues.

Among the remaining other traits, GWAS were performed for four meat quality traits: marbling, intramuscular fat, tenderness, and moisture percentage (Fig. S16A–G, Additional file 2). The results showed that only three SV loci were significant for intramuscular fat (Fig. S16B and Table 1), which were located in the intron regions of *HS3ST3A1*, *CFAP52*, and *STX8*. Heart, liver, and lung weight were also investigated for their associations with SVs, identifying one significant locus in each of the heart (Fig. S16E) and liver weight (Fig. S16F), which were a 2,681-bp DEL overlapping 3 bp with the *NOL10* exon and a 278-bp DEL upstream of *MRS2*, respectively. Considering that a certain tolerance is needed for the determination of SV breakpoint locations [81], the exonic variants still need further validation.

## Discussion

Here, we performed an SV study based on a resource population constructed from Large White and Min pigs. A typical approach to SV research is to take the results of multiple software intersections to improve the accuracy when identifying variants. This strategy has been reported to not reliably improve performance and in some cases even aggravate false discoveries [22]. Therefore, merging the analysis results of several software instead of including only overlapping regions is expected to maximize the performance of each software, which improves the sensitivity of SV identification to obtain more new SV loci. Using this approach, we developed a high-quality SV map with 53.95% newly discovered SV loci compared to the Ensemble public SV database, which will greatly enrich the public SV database. Then, the genotyping accuracy of the SV loci was validated by PCR to be more than 94%. We suggest that it will be necessary to perform multiple SV software in future studies and retain specific results from each software which will not only allow for the identification of more new SV loci, but also maintain accuracy.

Generally, when constructing a segregating population, high depth sequencing is allocated to the parental generation and low depth sequencing to the F2 generation or even more distant generations for cost reasons. In contrast, low coverage sequencing for SV identification appears to reduce sensitivity and accuracy. Here, we designed a novel approach for population genetic studies, which identified reliable SV loci in F0 individuals at high sequencing depths and then used these loci to genotype the F2 population according to the corresponding genomic positions for GWAS to identify causal SV loci due to breed differences. Our results show that almost all loci were successfully genotyped, confirming the reliability of this approach. This approach improves the accuracy of SV identification and increases the efficiency of the analysis by avoiding the identified variants in a large population. The analysis detected genes identified in previous studies using SNP markers including *ILRUN*, *TFEB*, *RCAN2*, and *VRTN* [72, 73, 79], which confirmed the accuracy of SV genotyping in the F2 population and the potential of SVs as markers. To our knowledge, this is the first report of this method in livestock studies. In addition, third-generation sequencing has begun to be applied in the study of animal and plant genomes, which has more potential to identify larger structural variants. However, its application is limited due to its high cost, especially within large populations. Genotyping in second-generation sequencing data using SV loci identified by third-generation sequencing data is a potential solution, although the reported recall of genotyping is currently only about 50% [82].

Compared to SNPs, SVs in non-coding regions are more likely to alter gene expression and phenotype through dosage effects, and SVs can also modify expression levels by directly altering gene copy numbers [83–86]. Therefore, using SVs as markers for directly performing GWAS is expected to identify causal loci affecting phenotypes. There have been several previous studies on SVs in the pig genome, but SV-based GWAS in the pig genome have been rarely reported [13–15]. The Large White × Min pig resource population provides an opportunity to deepen the understanding of the potential and biological role of SVs as markers for association studies. Moreover, insertional variants in particular have rarely been included in SV studies due to their complex genotyping process, and the phenotypic impact of insertional variants remains largely unknown. While transposon insertion identification depends on reference sequences, which facilitates subsequent genotyping, and a previous study has confirmed that approximately 80% of the variation in the pig genome overlaps with transposable elements [15], which provided an opportunity to investigate the contribution of insertional variants to the phenotype in the present study. Therefore, we performed GWAS on four SV types in the pig genome, providing new insights into the contribution of different SV types to the phenotype. To our knowledge, this is perhaps the most comprehensive SV-based GWAS of the pig genome to date.

Bone is a highly complex and active mineralized material. Bone tissue undergoes a continuous cycle of osteoclast bone resorption and osteoblast bone formation [87] and then receives mechanical loads from the musculoskeletal system and interactions with other biological systems (such as the endocrine, nervous, and immune systems) in order to maintain the shape, volume, and density of the bone [88]. During the growth period, there is intense bone formation to increase body size [89]. In this study, we performed GWAS and revealed a large number of candidate genes associated with skeleton in pigs. Among them, *FKBP5* and *MRS2* are involved in the differentiation and formation of osteoclasts, respectively. In contrast, *TFEB* and *RCAN2* are associated with the differentiation and function of osteoblasts. We hypothesize that, during domestication and selection, these key candidate genes may be affected due to surrounding or internal SVs regulation, resulting in differential body size between breeds. Based on previous studies, SV in the intron region can cause alternative splicing of RNA [90, 91] and play a promoter or enhancer role [92, 93], whereas SV located upstream or downstream of a gene may be associated with transcriptional regulation of that gene, especially transposon insertions, which have been reported to play a functional role in carrying cis-regulatory elements [94–97].

## Conclusion

In this study, we constructed a high-quality SV map using high-coverage resequencing data from the Large White and Min pigs. More than half of the SV loci were reported for the first time by merging the results of 5 SV tools, suggesting the need to use multiple software for SV analysis and to retain the specific variants identified by different SV tools. GWAS for 36 traits showed that SVs were mainly associated with skeletal size, which may contribute to the differences in body size between European and Chinese pig breeds.

### Supplementary Information

**Additional file 1: Table S1.** Sample information. **Table S2.** TE reference sequence. **Table S3.** Primers for 1–10 Mb SV validation. **Table S4.** Primers for SV genotype accuracy validation. **Table S5.** Number of all SV types identified per individual. **Table S6.** SV distribution for different length ranges. **Table S7.** Information on SV loci annotated as HIGH impact. **Table S8.** Top 5% fixed index (*F*_ST_) statistics based on Weir and Cockerham. **Table S9.** Primers for significant loci of skeletal-related traits.

**Additional file 2: Fig. S1.** Schematic design of SV primers for 1–10 Mb. **Fig. S2.** Schematic diagram of differential genotype screening. **Fig. S3.** SV Pipeline. **Fig. S4.** Validation of DEL variants at 1–10 Mb loci. **Fig. S5.** Validation of DUP variants at 1–10 Mb loci. **Fig. S6.** Validation of INV variants at 1–10 Mb loci. **Fig. S7.** The accuracy of SV genotyping was verified by agarose electrophoresis. **Fig. S8.** Venn diagram of SV loci in public databases and this study. **Fig. S9.** PCA plot of the 50k chip in 19 F0 individuals. **Fig. S10.** KEGG pathway. **Fig. S11.** Validation of SVs involving carcass traits using Sanger sequencing. **Fig. S12.** Manhattan plots for scapular width (**A**), chest width (**B**), chest depth (**C**), abdominal circumference (**D**), waist width (**E**), hip width (**F**), hip length (**G**), and hip circumference (**H**). **Fig. S13.** Manhattan plots for total weight of front bone (**A**), total weight of middle bone (**B**), total weight of hind bone (**C**), scapula length (**D**), humerus length (**E**), forearm bone length (**F**), hip bone length (**G**), femur length (**H**), calf bone length (**I**), and vertebral number (**J**). **Fig. S14.** Venn diagrams of three bone weight and six bone length traits overlapping with carcass length, body length, body height, cannon circumference, and bone rate. **Fig. S15.** Manhattan plots for total weight of front lean meat (**A**), total weight of middle lean meat (**B**), total weight of hind lean meat (**C**), total weight of front fat (**D**), total weight of middle fat (**E**), and total weight of hind fat (**F**). **Fig. S16.** Manhattan plots for marbling (**B**), tenderness (**C**), moisture percentage (**D**), heart weight (**E**), liver weight (**F**), and lung weight (**G**).

## Data Availability

The sequencing data of the F0 and F2 individuals used in this study have been submitted to the Genome Sequence Archive (GSA) with the accession number CRA002451.

## Abbreviations

DEL: Deletion
DUP: Duplication
GWAS: Genome-wide association studies
INV: Inversion
MEI: Mobile element insertion
SNP: Single nucleotide polymorphism
SV: Structural variation
