# Supplementary material for: Associations of genome-wide structural variations with phenotypic differences in cross-bred Eurasian pigs
Source: J Anim Sci Biotechnol. 2023 Oct 7;14:136. doi: 10.1186/s40104-023-00929-x (PMC10559557; doi:10.1186/s40104-023-00929-x)
Supplement: Supplementary file 2 — Additional file 2: Fig. S1. Schematic design of SV primers for 1–10 Mb. Fig. S2. Schematic diagram of differential genotype screening. Fig. S3. SV Pipeline. Fig. S4. Validation of DEL variants at 1–10 Mb loci. Fig. S5. Validation of DUP variants at 1–10 Mb loci. Fig. S6. Validation of INV variants at 1–10 Mb loci. Fig. S7. The accuracy of SV genotyping was verified by agarose electrophoresis. Fig. S8. Venn diagram of SV loci in public databases and this study. Fig. S9. PCA plot of the 50k chip in 19 F0 individuals. Fig. S10. KEGG pathway. Fig. S11. Validation of SVs involving carcass traits using Sanger sequencing. Fig. S12. Manhattan plots for scapular width (A), chest width (B), chest depth (C), abdominal circumference (D), waist width (E), hip width (F), hip length (G), and hip circumference (H). Fig. S13. Manhattan plots for total weight of front bone (A), total weight of middle bone (B), total weight of hind bone (C), scapula length (D), humerus length (E), forearm bone length (F), hip bone length (G), femur length (H), calf bone length (I), and vertebral number (J). Fig. S14. Venn diagrams of three bone weight and six bone length traits overlapping with carcass length, body length, body height, cannon circumference, and bone rate. Fig. S15. Manhattan plots for total weight of front lean meat (A), total weight of middle lean meat (B), total weight of hind lean meat (C), total weight of front fat (D), total weight of middle fat (E), and total weight of hind fat (F). Fig. S16. Manhattan plots for marbling (B), tenderness (C), moisture percentage (D), heart weight (E), liver weight (F), and lung weight (G). [file 40104_2023_929_MOESM2_ESM.docx]

**
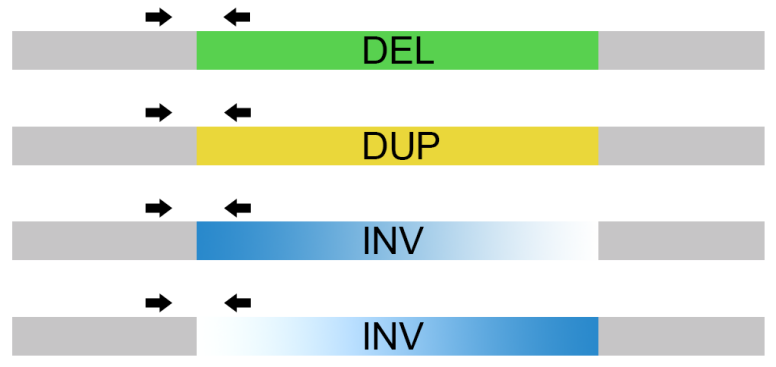
**

**Fig. S1** Schematic design of SV primers for 1–10 Mb. Different colors represent different types of variants, gray represents genomic sequences, and arrows represent the position of primers

**
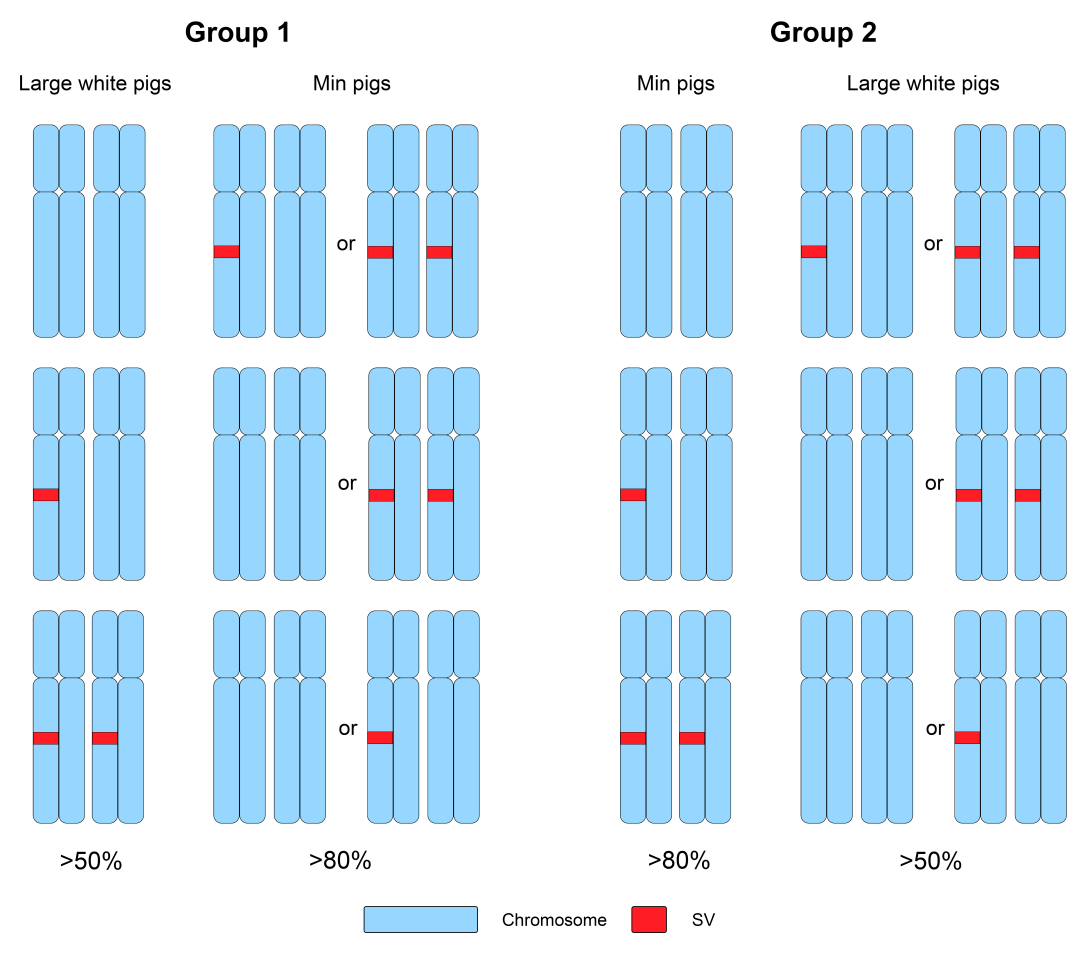
**

**Fig. S2** Schematic diagram of differential genotype screening. The screening strategy is to set a certain genotype of Large White or Min pigs, while the other breed appears with the remaining two genotypes. The numerals at the bottom of the graph represent the frequencies set for each genotype. Blue and red rectangles represent chromosomes and SVs, respectively


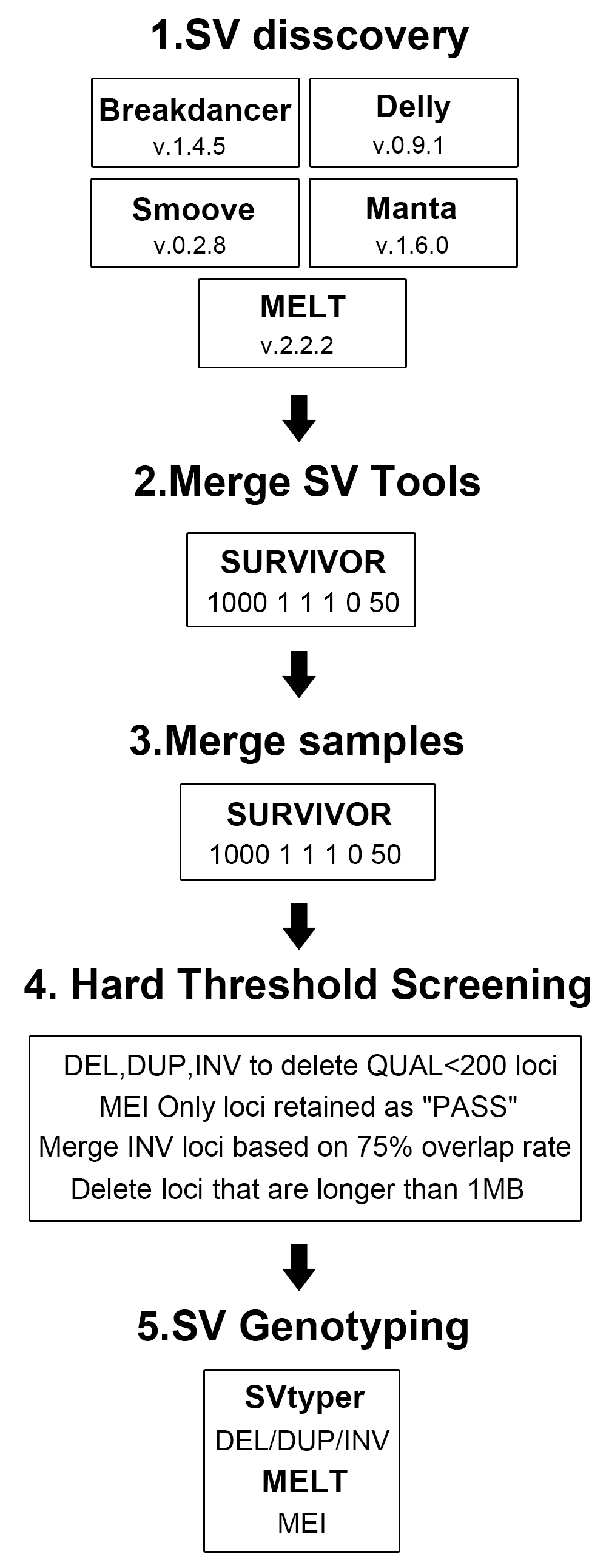


**Fig. S3** SV Pipeline. The SV pipeline is composed of several independent steps that run in a sequential manner. Step 1 shows the software and version used for SV discovery in this study. Steps 2 and 3 show the software and specific parameters for merging at the individual and population levels, respectively. Step 4 shows the filtering conditions for all SV loci. Step 5 describes the software required for genotyping the different SV types


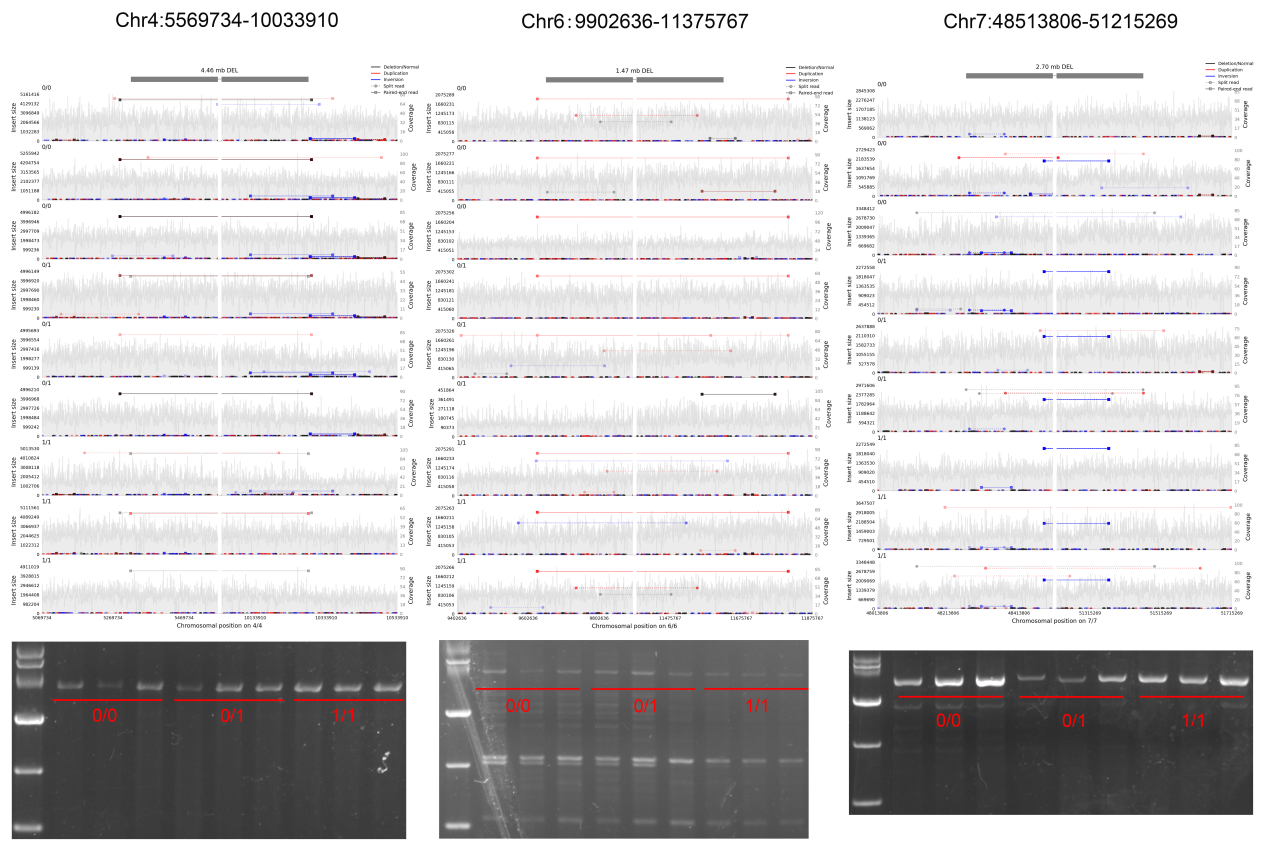


**Fig. S4** Validation of DEL variants at 1–10 Mb loci. The above plots were generated by visualizing the coverage, and the following electrophoresis plot was generated by setting primers based on the approximate 1500 bp position of the breakpoints around the DEL. TIANGEN marker IV was used as a DNA marker

**
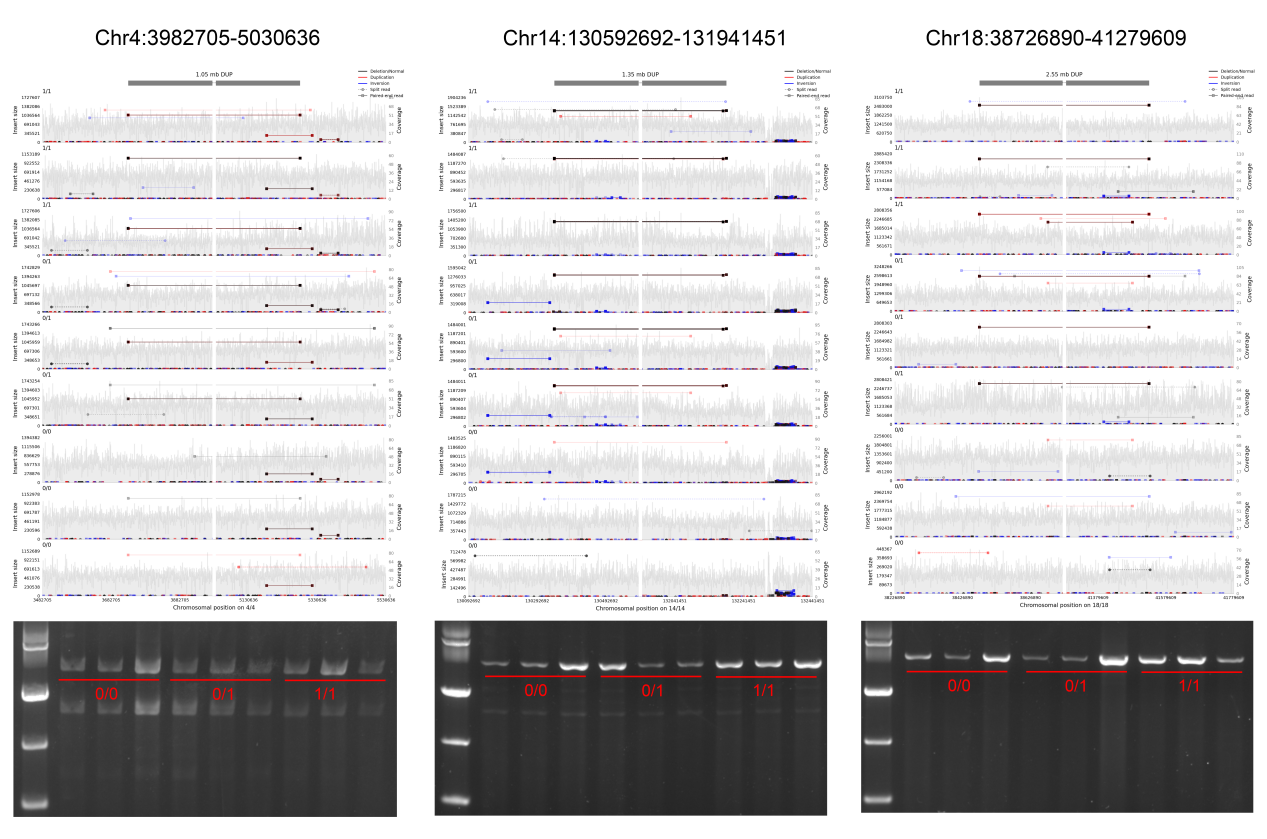
**

**Fig. S5** Validation of DUP variants at 1–10 Mb loci. The above plots were generated by visualizing the coverage, and the following electrophoresis plot was generated by setting primers based on the approximate 1500 bp position of the breakpoints around the DUP. TIANGEN marker IV was used as a DNA marker


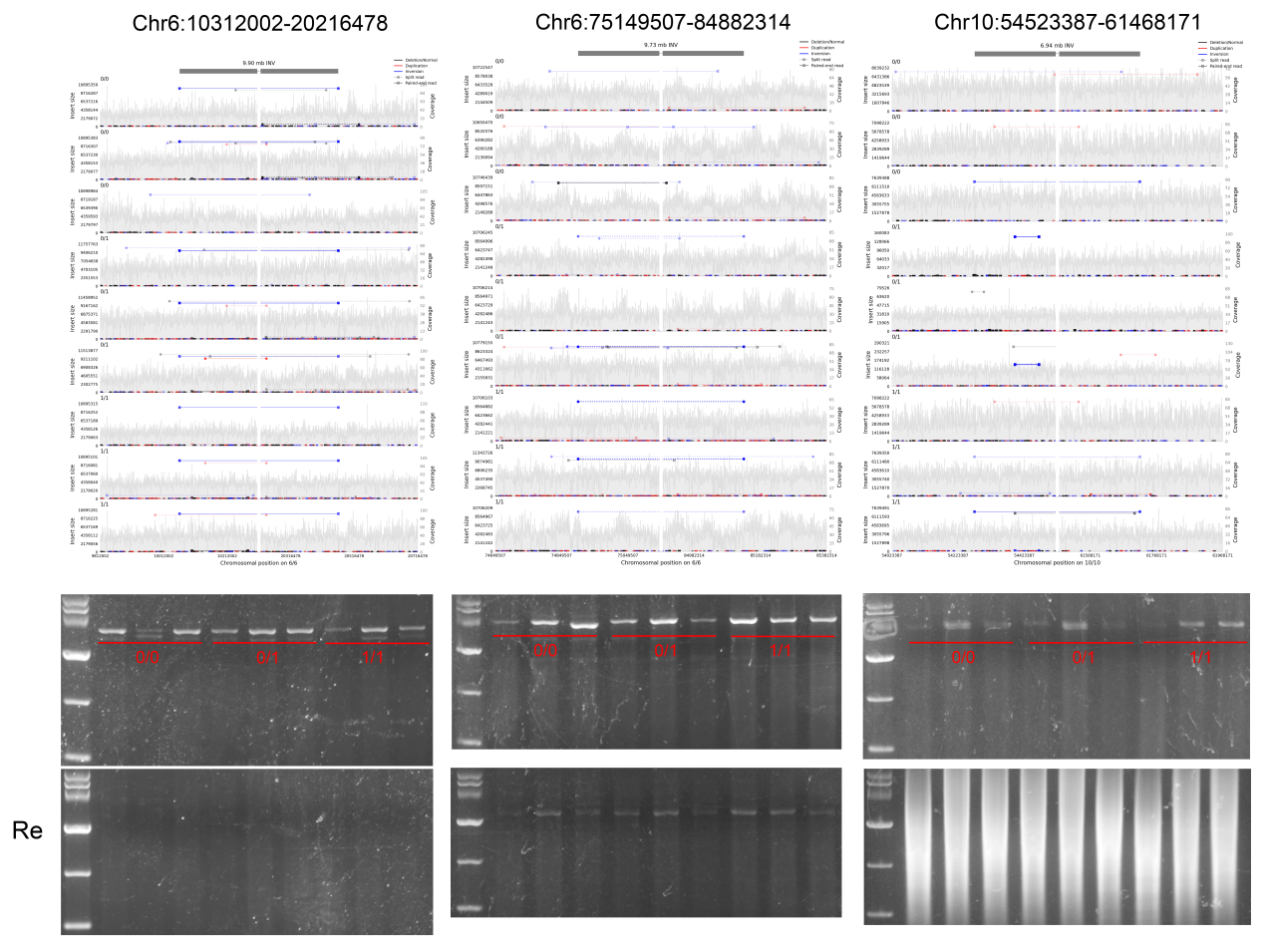


**Fig. S6** Validation of INV variants at 1–10 Mb loci. The above plots were generated by visualizing the coverage, and the following electrophoresis plot was generated by setting primers based on the approximate 1500 bp position of the breakpoints around the INV. TIANGEN marker IV was used as a DNA marker. Re: PCR amplification performed by reversing the sequence then designing primers


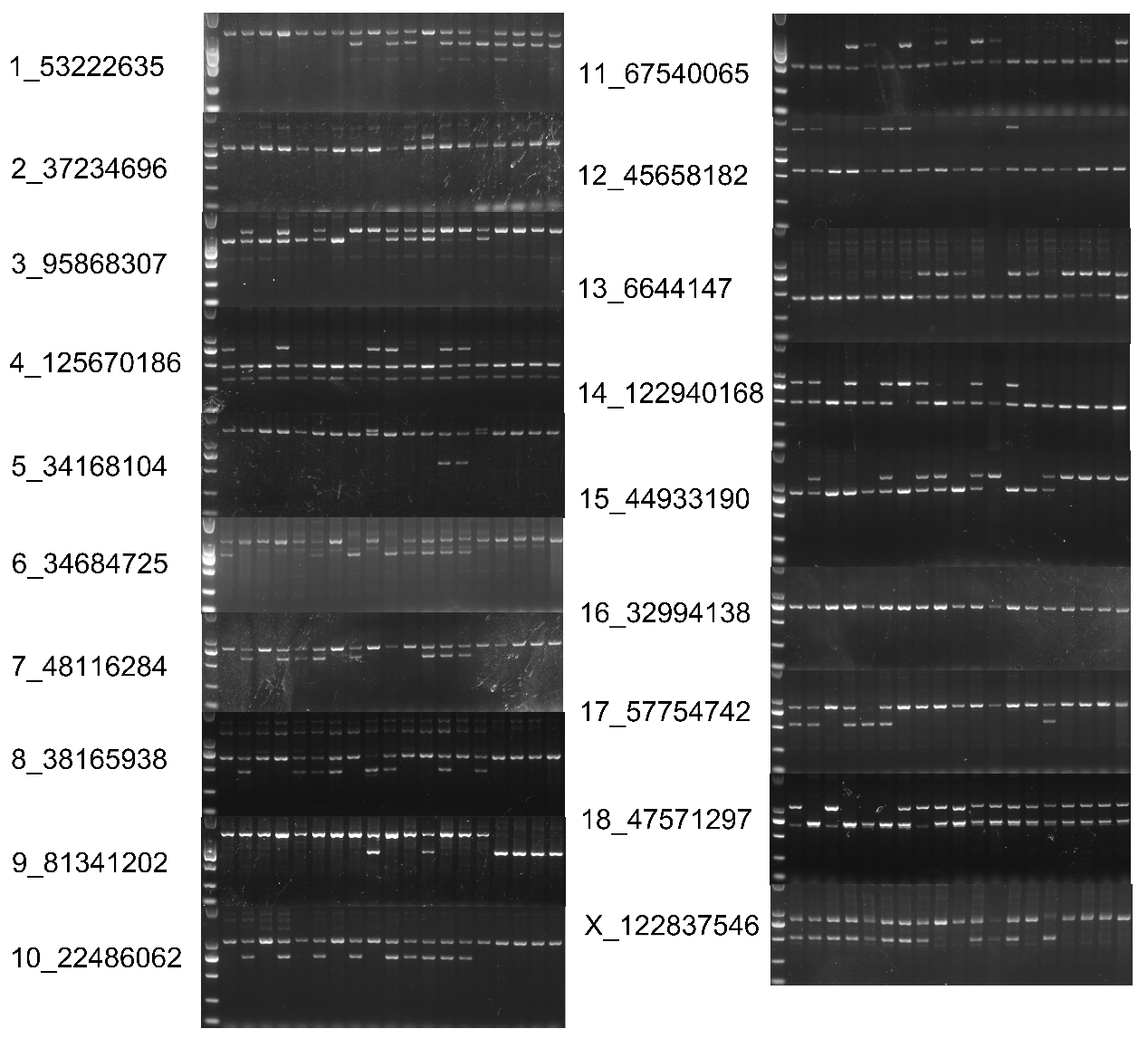


**Fig. S7** The accuracy of SV genotyping was verified by agarose electrophoresis. One locus per chromosome was randomly selected to verify the accuracy of the loci by PCR. The sample used 19 individuals from the F0 generation, the first 15 pigs were H1-H15 and the last four pigs were LW1–LW4. TaKaRa DL2000 was used as a DNA marker


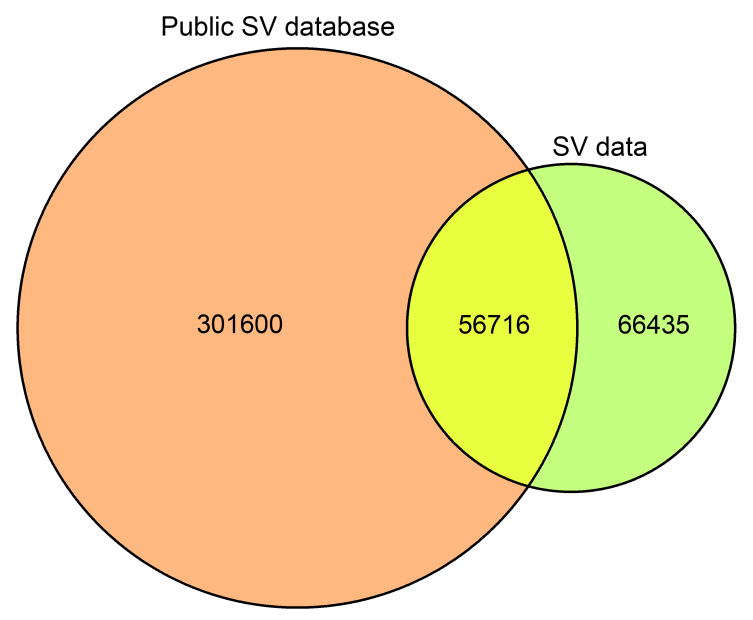


**Fig. S8** Venn diagram of SV loci in public databases and this study


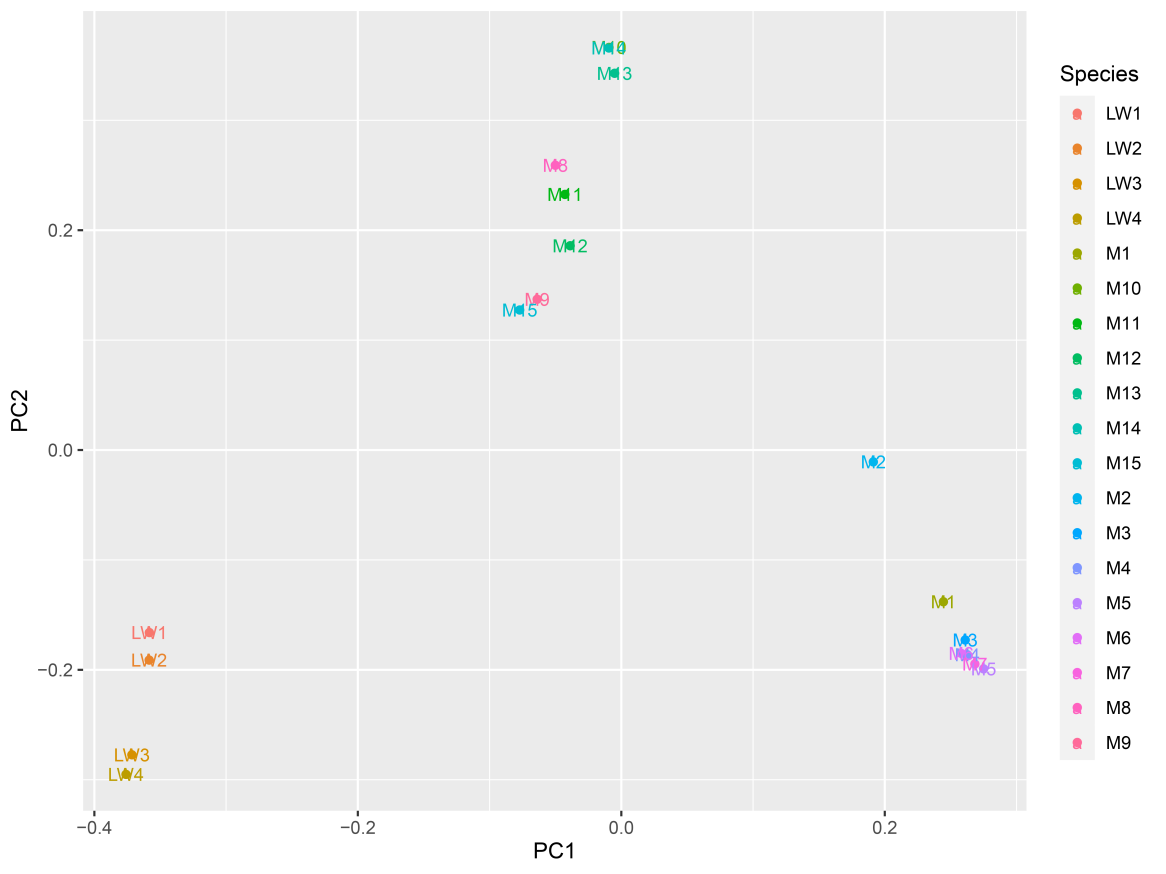


**Fig. S9** PCA plot of the 50k chip in 19 F0 individuals


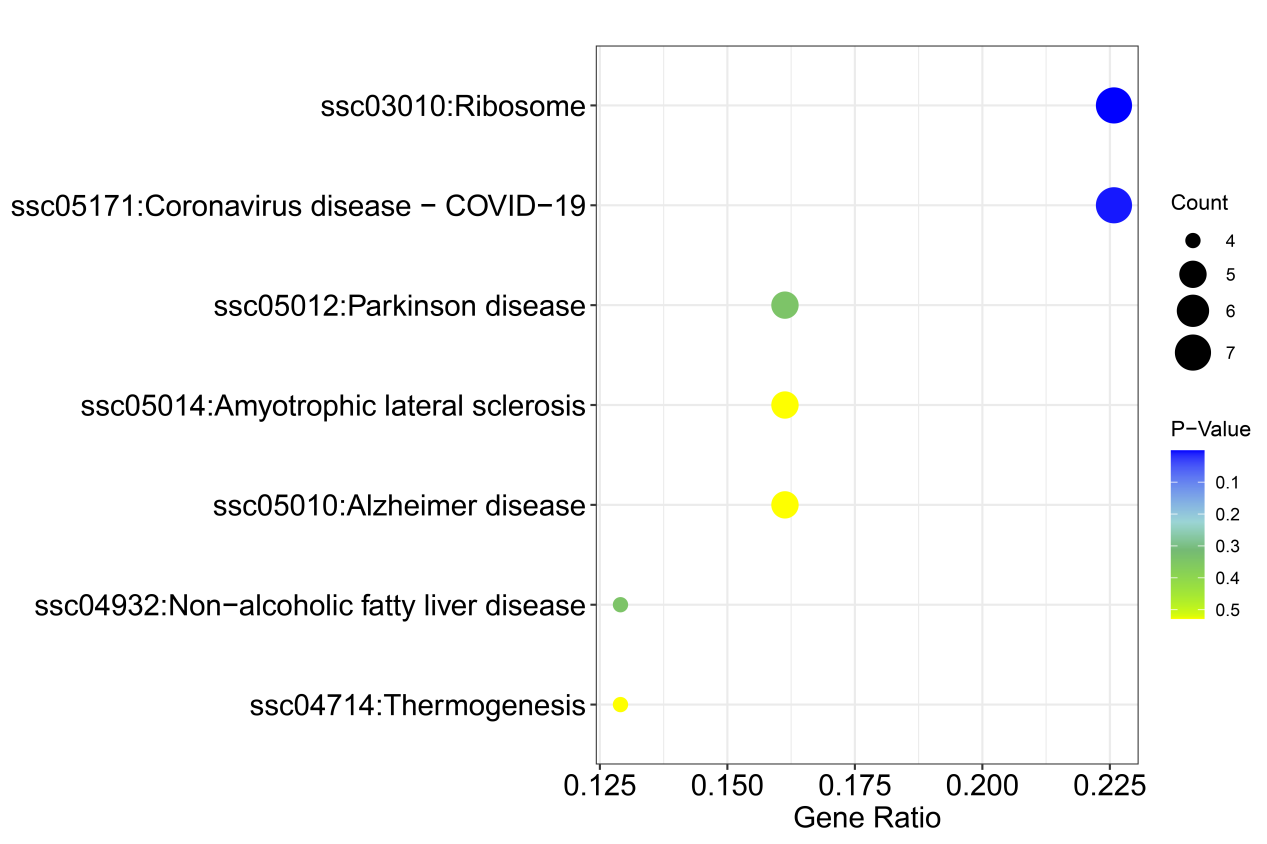


**Fig. S10** KEGG pathway. All "HIGH" effect SV loci containing 70 genes were performed for KEGG analysis and pathways with *P* < 0.05 were retained


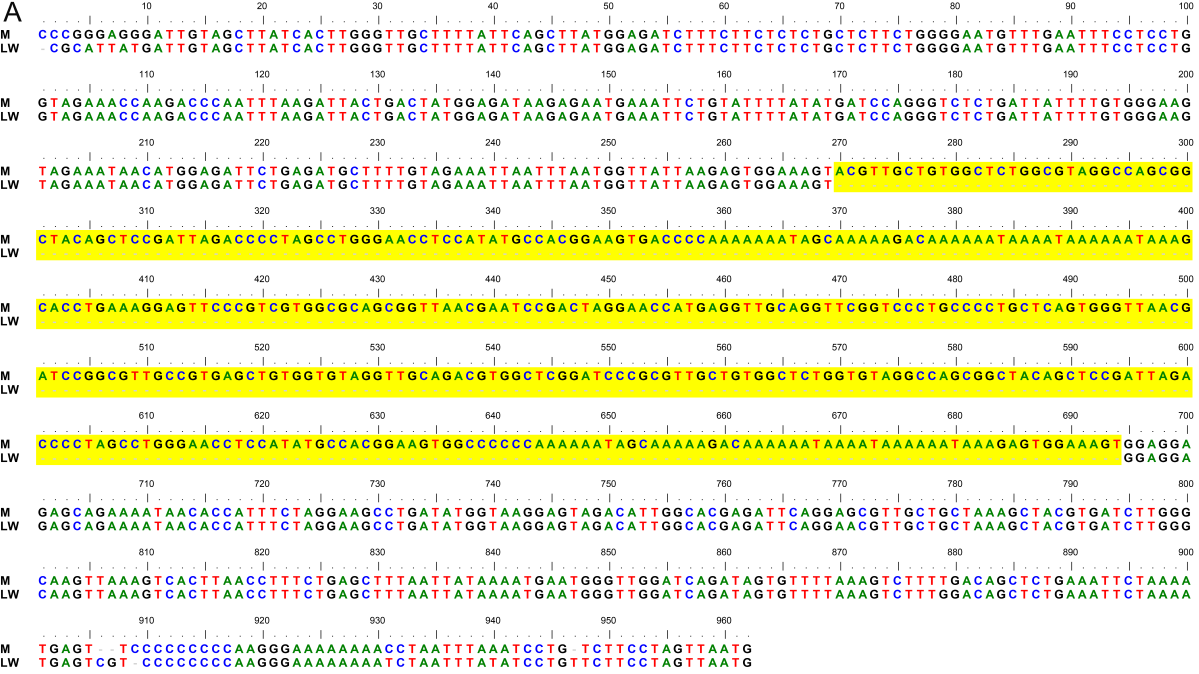

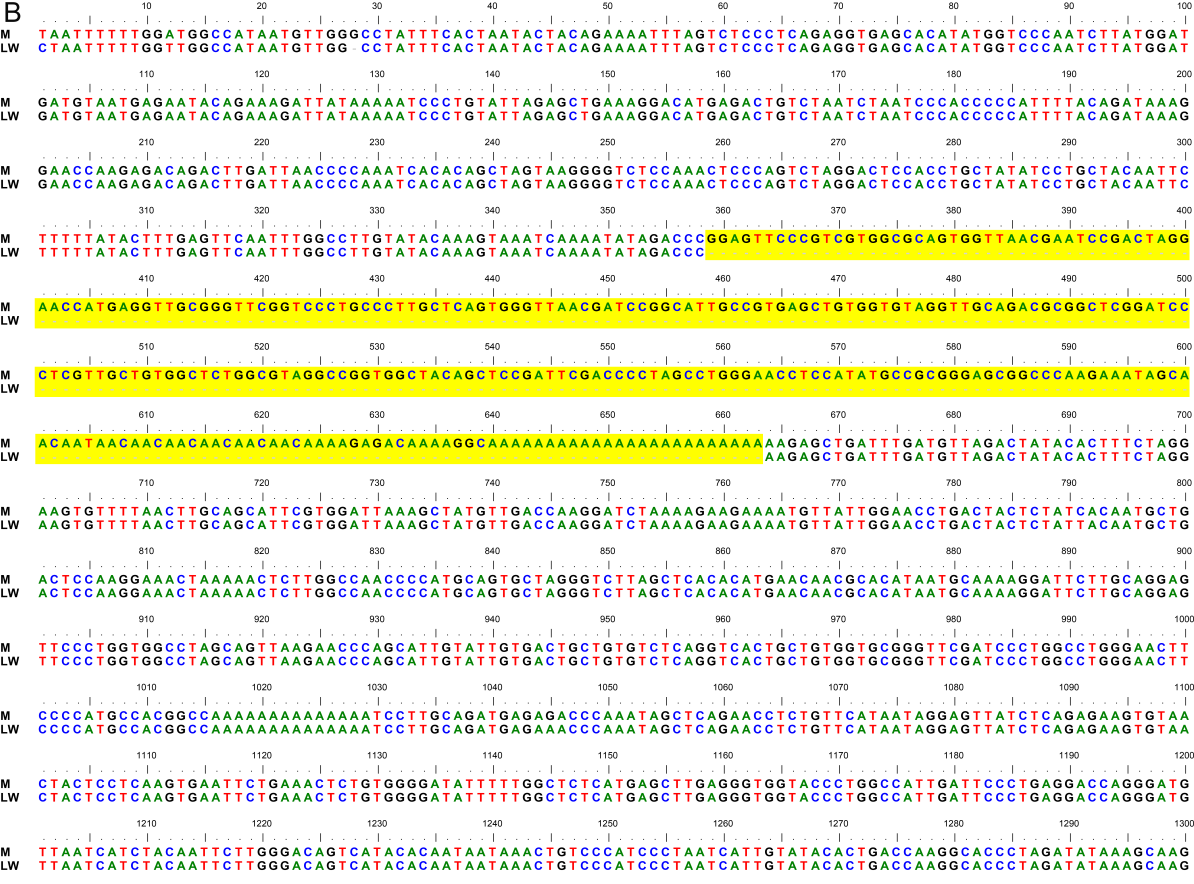

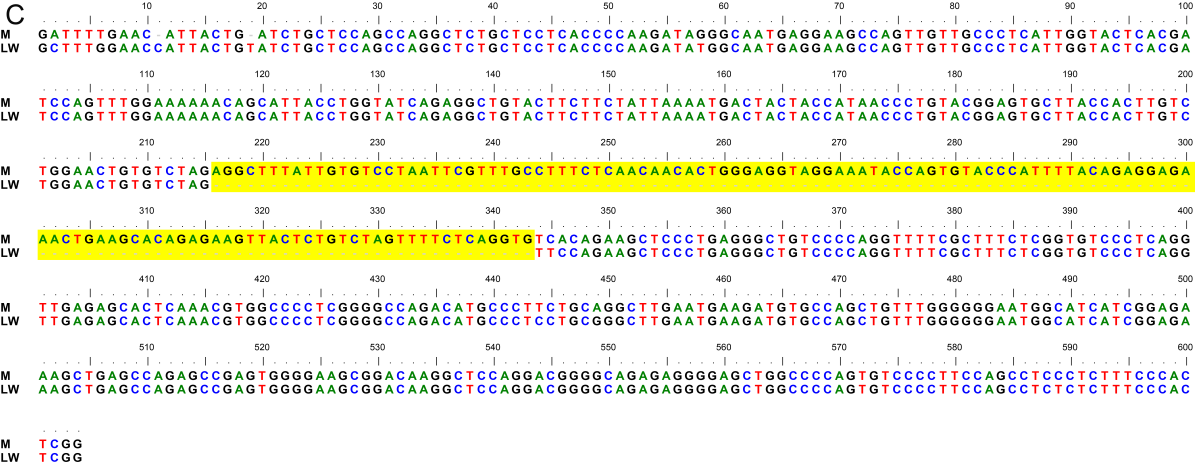

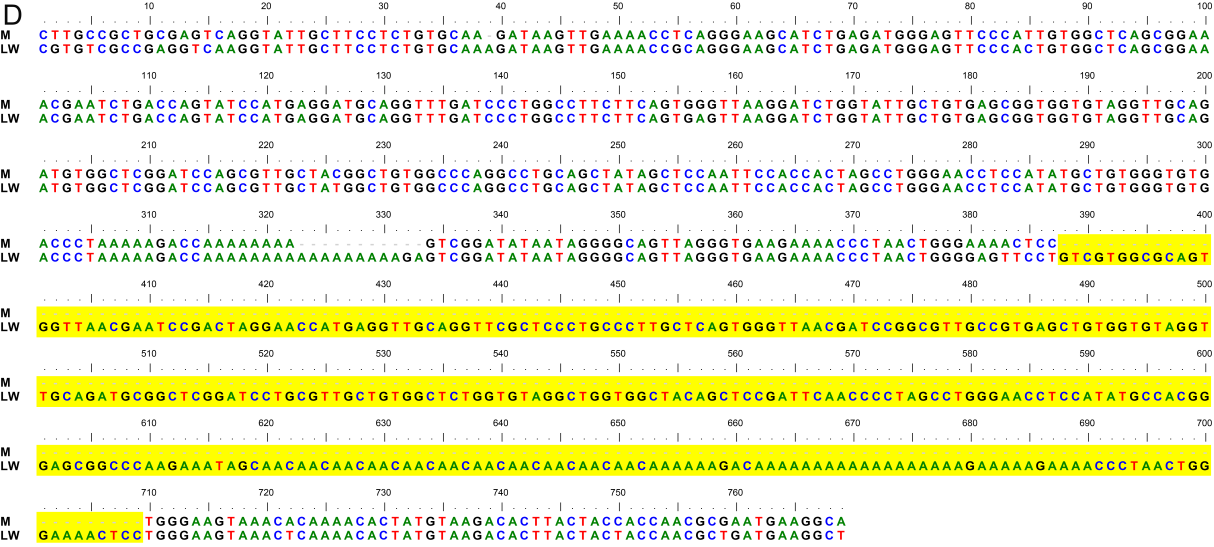

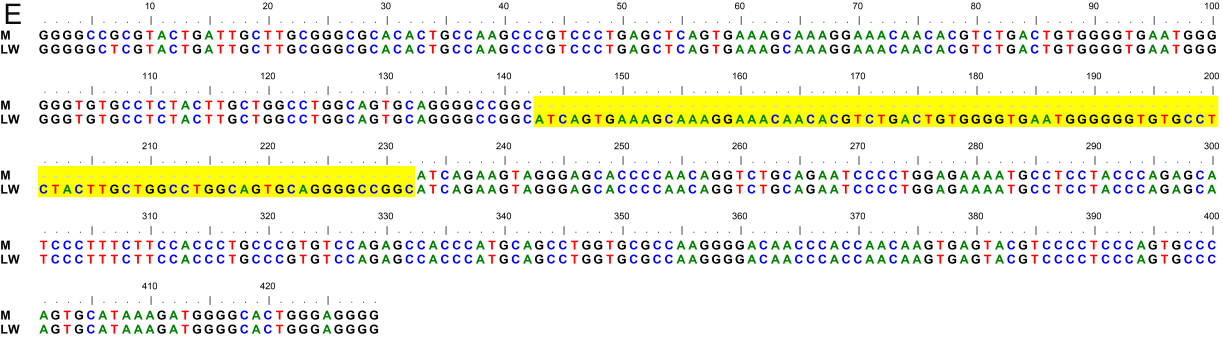

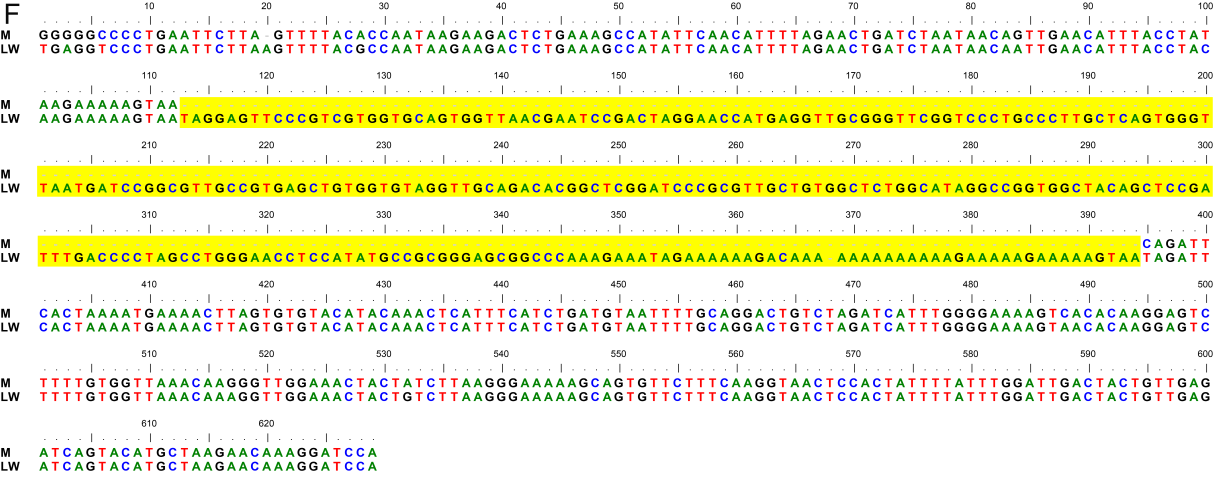

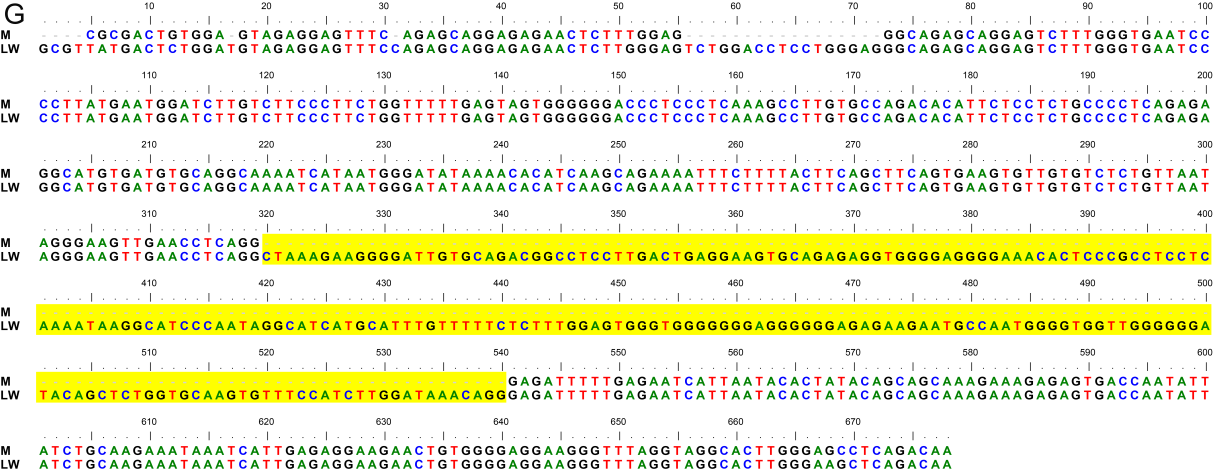


**Fig. S11** Validation of SVs involving carcass traits using Sanger sequencing. A Min pig and Large White pig with different genotypes were selected for Sanger sequencing and subsequently aligned. The seven genes are, in order, *FKBP5* (**A**), *ILRUN* (**B**), *TFEB* (**C**), *RCAN2* (**D**), *ANKS1A* (**E**), *MRS2* (**F**), and *GLP1R* (**G**)*.* The locations of the variants are marked using yellow rectangles

**
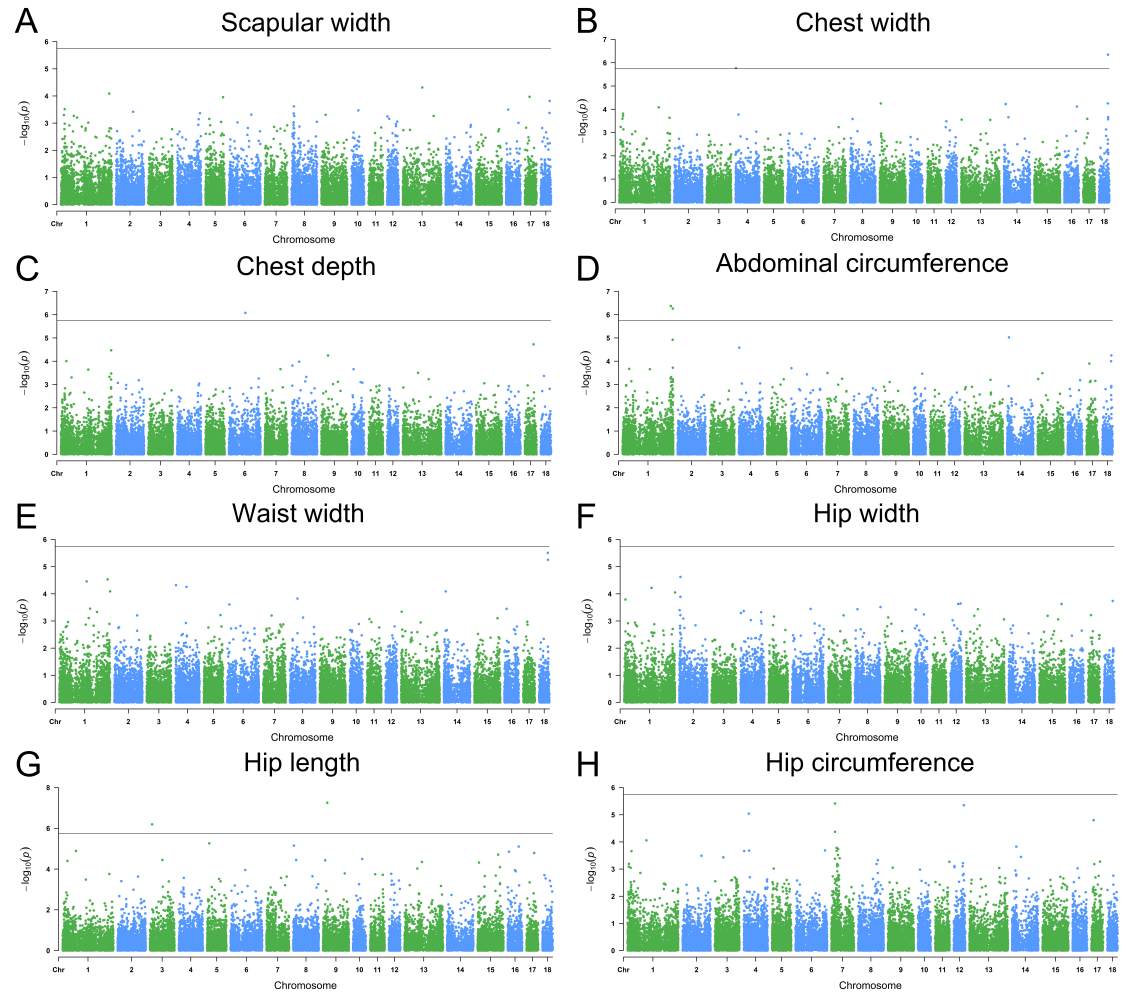
**

**Fig. S12** Manhattan plots for scapular width (**A**), chest width (**B**), chest depth (**C**), abdominal circumference (**D**), waist width (**E**), hip width (**F**), hip length (**G**), and hip circumference (**H**). The significance cutoff was defined as the Bonferroni test threshold, which was set as 0.05/(total number of SVs)


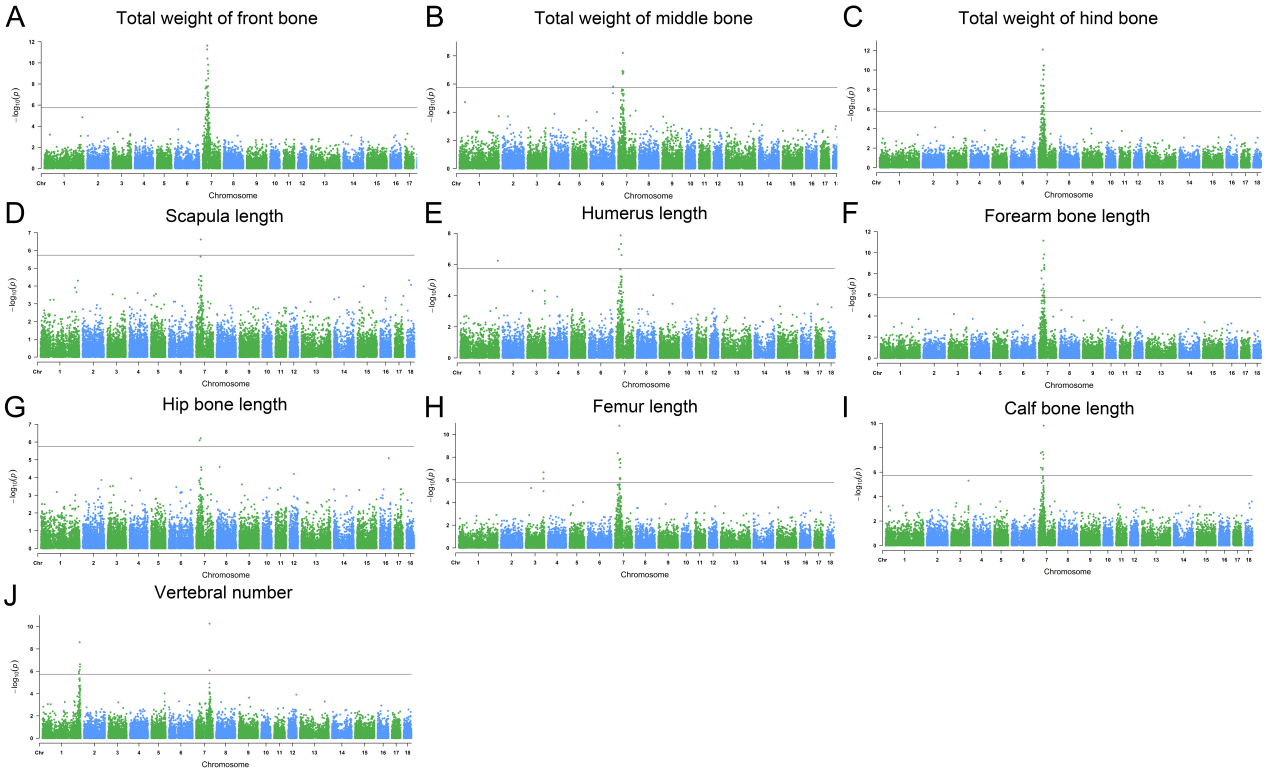


**Fig. S13** Manhattan plots for total weight of front bone (**A**), total weight of middle bone (**B**), total weight of hind bone (**C**), scapula length (**D**), humerus length (**E**), forearm bone length (**F**), hip bone length (**G**), femur length (**H**), calf bone length (**I**), and vertebral number (**J**). The significance cutoff was defined as the Bonferroni test threshold, which was set as 0.05/(total number of SVs)


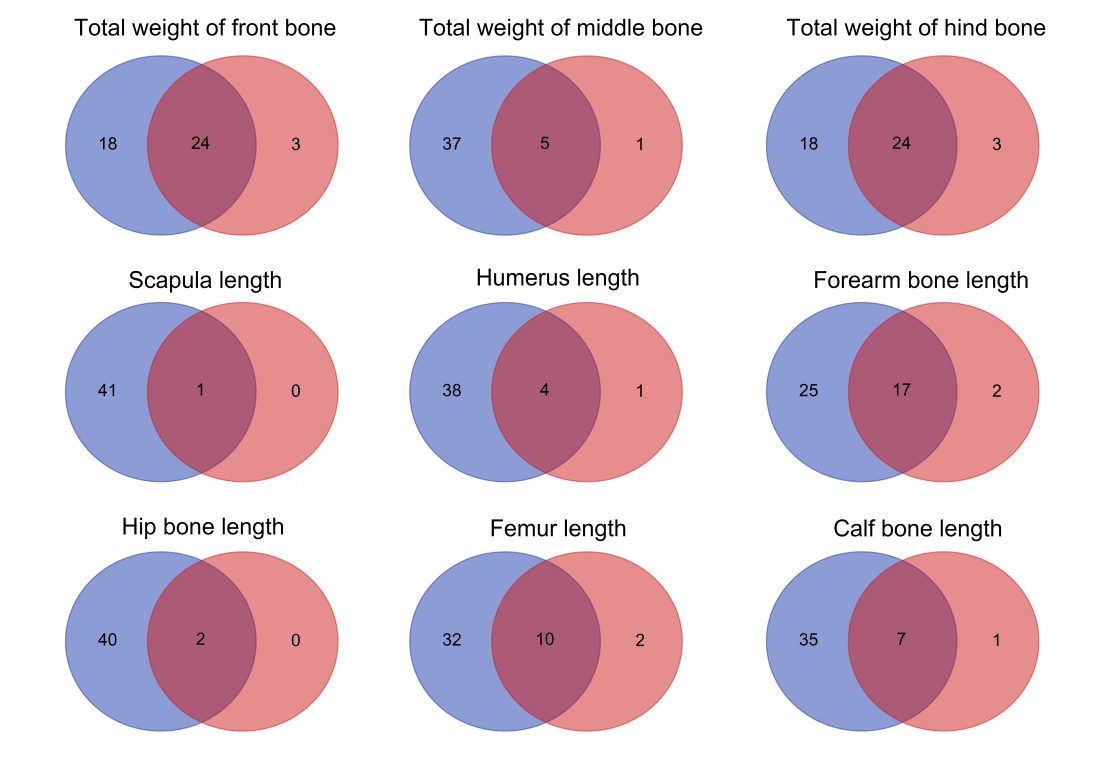


**Fig. S14** Venn diagrams of three bone weight and six bone length traits overlapping with carcass length, body length, body height, cannon circumference, and bone rate


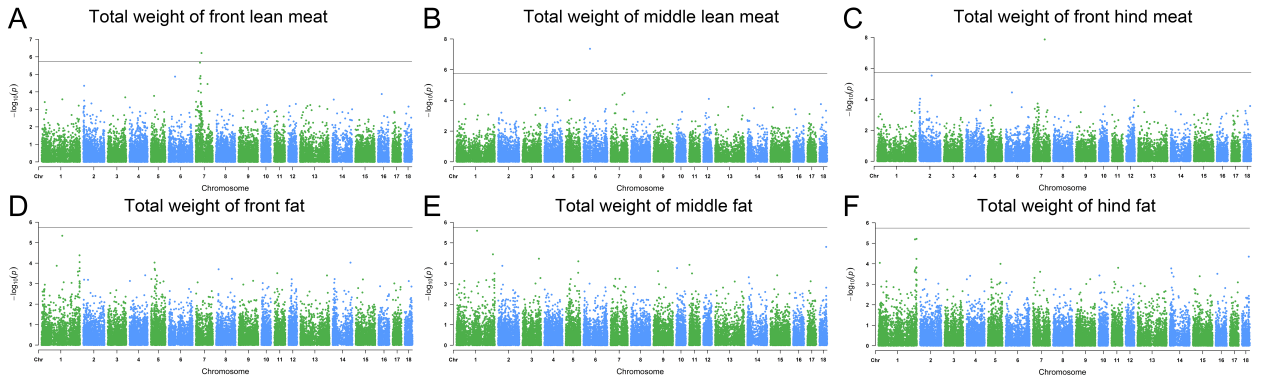


**Fig. S15** Manhattan plots for total weight of front lean meat (**A**), total weight of middle lean meat (**B**), total weight of hind lean meat (**C**), total weight of front fat (**D**), total weight of middle fat (**E**), and total weight of hind fat (**F**). The significance cutoff was defined as the Bonferroni test threshold, which was set as 0.05/(total number of SVs)


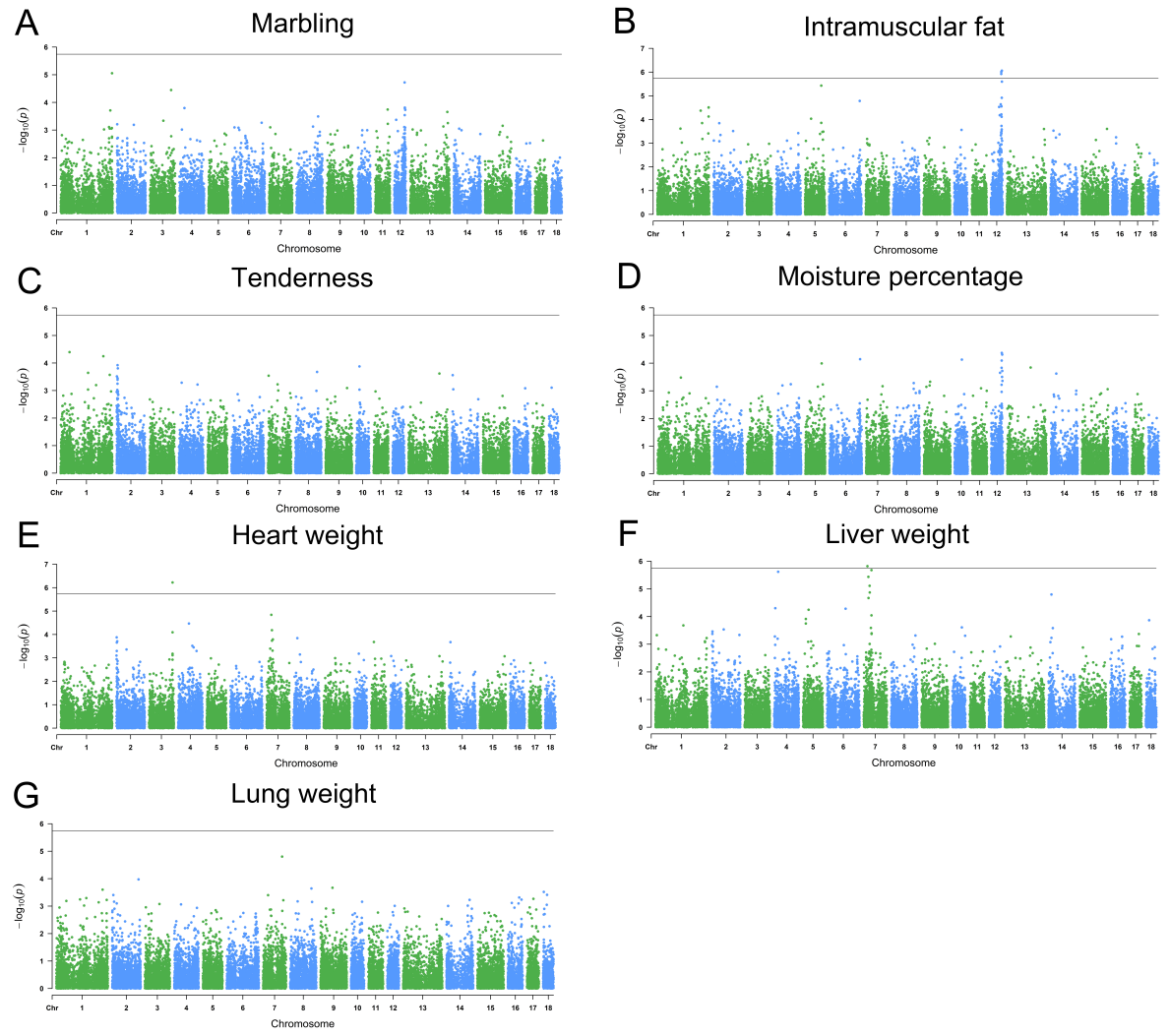


**Fig. S16** Manhattan plots for marbling (**A**), intramuscular fat (**B**), tenderness (**C**), moisture percentage (**D**), heart weight (**E**), liver weight (**F**), and lung weight (**G**). The significance cutoff was defined as the Bonferroni test threshold, which was set as 0.05/(total number of SVs)
